# DIALib-QC an assessment tool for spectral libraries in data-independent acquisition proteomics

**DOI:** 10.1038/s41467-020-18901-y

**Published:** 2020-10-16

**Authors:** Mukul K. Midha, David S. Campbell, Charu Kapil, Ulrike Kusebauch, Michael R. Hoopmann, Samuel L. Bader, Robert L. Moritz

**Affiliations:** Institute for Systems Biology, Seattle, WA 98109 USA

**Keywords:** Proteomics, Proteome informatics

## Abstract

Data-independent acquisition (DIA) mass spectrometry, also known as Sequential Window Acquisition of all Theoretical Mass Spectra (SWATH), is a popular label-free proteomics strategy to comprehensively quantify peptides/proteins utilizing mass spectral libraries to decipher inherently multiplexed spectra collected linearly across a mass range. Although there are many spectral libraries produced worldwide, the quality control of these libraries is lacking. We present the DIALib-QC (DIA library quality control) software tool for the systematic evaluation of a library’s characteristics, completeness and correctness across 62 parameters of compliance, and further provide the option to improve its quality. We demonstrate its utility in assessing and repairing spectral libraries for correctness, accuracy and sensitivity.

## Abstract (web summary)

Most data-independent acquisition (DIA) methods depend on mass spectral libraries for peptide identification but tools to assess library quality are lacking. Here, the authors develop DIALib- QC for the systematic evaluation and correction of spectral libraries.

## Introduction

Reliable identification and accurate quantification of peptides and proteins remains the central goal in mass spectrometry (MS) based proteomics^1,2^. Advancements in DIA methods have made them popular because of their increased coverage and unbiased MS/MS sampling of precursor ions detected or not, leading to more consistent peptide detection and quantitation across replicates^3–5^. The primary approach in DIA analysis requires prior knowledge of peptide fragmentation stored in spectral ion libraries^4,6–8^. As library quality directly influences DIA results, it is important to assess its characteristics and accuracy before drawing biological conclusions^8–10^. Several tools and pipelines are available to generate and merge spectral assay libraries, but few perform library quality assessment and are limited to a particular analysis pipeline, format or vendor, or are limited in scope, and none are able to correct a library. One such reported tool, SWATHXtend^9^, applies certain quality filters to generate clean hybrid libraries but does not evaluate spectral ion libraries for correctness. Here, our DIALib-QC software tool provides a path to ensure high-quality library usage for DIA analysis by evaluating and correcting spectral libraries for defects or biases (rather than blindly trusting and sharing for reuse). DIALib-QC performs an in-depth assessment, providing valuable characteristics of a library and also enables users to correct and generate an error-free library by eliminating problematic assays. These DIALib-QC features are extremely relevant to all DIA users regardless of the pipeline used (whether generating in-house libraries or using public repository libraries), library formats, and DIA analysis tools. DIALib-QC reports a tabular and graphical summary of common DIA library formats including OpenSWATH, Spectronaut, PeakView, and Prosit (generic text, Spectronaut compatible). We employ DIALib-QC and targeted data extraction with library-based DIA tools, PeakView and Spectronaut^11^ to demonstrate the importance of high-quality spectral libraries and their impact on DIA results. Further, we show DIALib-QC and its functionality to repair an ion library by correcting mass errors (recomputing to theoretical *m/z* values) and removing conflict assays to generate a clean and correct spectral library for DIA analysis. DIALib-QC is freely available to download and also integrated within the SWATHAtlas (http://www.swathatlas.org) interface, the free, extensively-used spectral ion library repository for DIA analysis, where libraries submitted by users are made accessible for reuse, thereby eliminating the effort of generating complex DIA spectral libraries a priori. We recommend assessing and correcting spectral libraries using DIALib-QC, prior to DIA analysis, to ensure correct identification and accurate quantitation of peptides and proteins from DIA data processing tools.

## Results

### Characteristics of the DIALib-QC tool

**Fig. 1:**
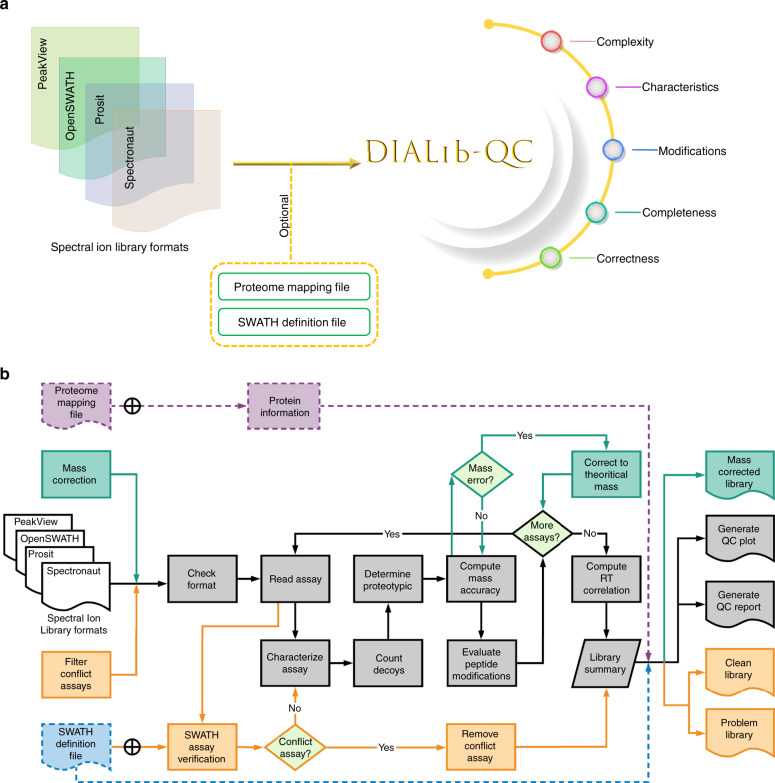
Concept and process of DIALib-QC tool. **a** The software accepts ion libraries of different formats and optional SWATH definition and proteome mapping files as input. DIALib-QC assesses DIA ion libraries based on 62 criteria that can be broadly organized in five categories. The correctness category can further be used for repairing an ion library. **b** The flowchart describes the process of the DIALib-QC tool, available at www.swathatlas.org. The workflow in black solid lines shows all functions to analyze an assay library and generates an assessment report and graphical summary plots. Mass correction (green solid lines) and filtering conflict assays (orange solid lines) functions are tandem features that generate a new library. Using the mass correction parameter results into a mass corrected library while using the filter conflict assays parameter segregates the query library into a problem (containing only conflict assays) and a clean (error-free) library. The proteome mapping file (purple dashed line) and SWATH definition file (blue dashed line) are optional input files which provide information related to peptide/protein characteristics and help to asses conflict assays, respectively. DIALib-QC evaluates systematically different library characteristics and generates a report highlighting attributes that may impact the identification and quantification of proteins.

DIALib-QC inspects 62 criteria of a library and provides valuable insights of its composition in a tabulated form. These criteria are broadly organized into five categories (Fig. 1a, Supplementary Data 1) and are processed as depicted in Fig. 1b. These include (i) complexity of the library (number of peptides, peptide ions, and fragment ions); (ii) characteristics of the library that are physical attributes of precursor ions (e.g., charge distribution, retention time (RT) information, and properties of the enzymatic digestion) and fragment ions (e.g., number of fragments per precursor, ion series, and intensity distribution); (iii) modifications describing the number and types of posttranslational modifications in the library; (iv) completeness reflecting how comprehensively the library covers the target proteome, and percentage of proteotypic assays versus shared proteins; and (v) correctness highlighting diagnostic criteria that should be carefully evaluated before DIA analysis. DIALib-QC reports on these attributes as they are frequently encountered in poorly constructed libraries and as yet there has been no tool to interrogate them. Of the five, we focus hereafter on the correctness category that highly influences the DIA analysis.

1. Target/decoy percentage: DIALib-QC reports the percentage of target, mixed (map to both target and decoy), and decoy assays present in the library. Decoy assays are assays to peptides that are not present in the target proteome. These assays could be in silico derived, spiked into the library or inherited from DDA analysis during library generation. Some DIA pipelines require decoy assays for FDR estimation using a target-decoy approach, provided sufficient decoy assays are present for robust error modeling^12^.
2. Mass accuracy: DIALib-QC compares precursor and fragment ions *m/z* to theoretical values and provides the average delta mass, highlighting the accuracy of masses used in the library. The total number of these erroneous assays along with assays that cannot be verified with the input SWATH definition file (see category 4 below and Methods) are reported as the criterion problem assays. Such assays can be further explored in the DIALib-QC ion library repair feature to generate a clean library, free from mass errors, and conflict assays.
3. RT correlation fit: DIALib-QC estimates the RT fit by comparing the RT of [M + 2H]^2+^ and [M + 3H]^3+^ charge states of the same peptide. This assesses the chromatography and RT normalization, based on reference peptides in the library. Poor RT correlation can lead to decreased identifications, due to reduced selectivity of DIA analysis, as chromatogram extraction occurs in a RT window centered around the expected elution time for the queried peptide^6^.
4. DIA verification: DIALib-QC reports the number of precursor ions that have at least one conflict fragment ion, and the total number of fragment ions that fall into the same DIA bin as the parent precursor ions. Such conflict assays may cause erroneous quantitation as un-fragmented precursor ion signals contribute to fragment ion intensities, thus overestimating peptides.

We sequentially explored these diagnostic criteria by explicitly generating defective ion libraries to demonstrate the DIALib-QC tool performance and effects on DIA analyses. We generated four spectral ion libraries from: (a) SWATHAtlas (pan-human library (PHL)) perturbed explicitly in q3 *m/z* value; viz. good (control), q3bad_0.01, q3bad_0.02, and q3bad_0.05, and seven sample-specific spectral ion libraries from (b) in-house K562 cell line, perturbed implicitly; viz. base, conflict, good (control), q3bad, raw, q3bad_mz_corrected, and q3bad_mz_corrected_clean (see Methods, Supplementary Fig. 1). All eleven libraries were assessed by DIALib-QC and their reports are provided in Supplementary Data 2.

### Systematic quality evaluation of spectral ion libraries

**Fig. 2:**
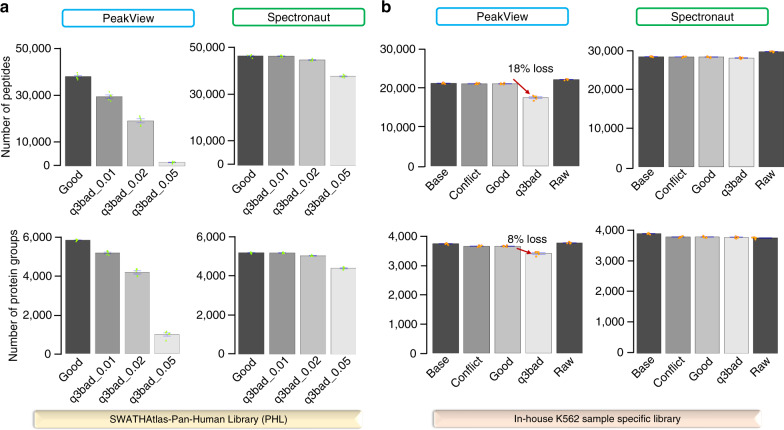
Comparative performance of spectral libraries analyzed in DIA tools. **a** SWATHAtlas—pan-human library (PHL). DIALib-QC identifies the q3 mass error in different PHL libraries. Despite of the q3 *m/z* error in the libraries, Spectronaut provides higher sensitivity than PeakView as it implements a MS2 calibration strategy. Both peptide and protein group identifications decrease with increasing q3 mass error with PeakView. **b** In-house K562 sample-specific library. DIALib-QC evaluates various diagnostic criteria and their effect on the number of identifications is represented with different in-house K562 sample-specific libraries. The bar plots highlight the effects of decoy assays (base library), conflict assays (fragment ion within the parent q1 precursor swath bin), fragment ion mass error, and redundant spectra on the SWATH analysis. The number of peptides and proteins identified at an estimated 1% FDR were similar regardless of the library used (except q3bad) and software used (PeakView or Spectronaut.) The fragment ion mass error in the q3bad library had the greatest effect on the PeakView performance, decreasing peptide, and protein identifications by 18% and 8%, respectively, compared to the good library (marked by red arrows). The error bars indicate the variability within five replicates represented as standard error of the mean. These are calculated as the ratio of standard deviation of the number of quantified peptides or proteins observed in each gradient replicate to the square-root of the sample size (*n* = 5). The small green and orange dots denote the number of identifications in each replicate in the PHL and the in-house K562 sample-specific library, respectively. The bar graphs were generated with data provided in Supplementary Data file 4, 5.

Analysis of PHL and K562 good libraries by DIALib-QC shows >0.99 RT correlation (R-squared value), indicating high similarity between +2 and +3 charge states of a same peptide (Supplementary Fig. 2), and confirming high-quality libraries in the present DIA analysis. DIALib-QC identified the average *m/z* difference between the q3 *m/z* value in the library and the theoretical *m/z* value for each peptide sequence. Because these values may be computed differently from various software tools, or empirically derived, even small mass differences can have dramatic effects on DIA analysis. Specifically, DIALib-QC was used to assess average fragment ion *m/z* differences that increased from 0 *m/z* to 0.05 *m/z* across four PHL spectral libraries (see Methods). DIA analysis with PeakView resulted in a decrease of identified peptides from 38,944 to 878, and protein groups from 5801 to 653 (Fig. 2a). Increased fragment ion mass error decreased DIA sensitivity, even when those errors were small as reflected in their score distribution of target and decoy assays (Supplementary Fig. 3). Using the same libraries to analyze the data with Spectronaut, peptide and protein groups identification decreased from 48,947 to 37,932 and 5347 to 4276, respectively (Fig. 2a). Here, Spectronaut is less affected by fragment ion mass errors than PeakView because it implements a preprocessing MS2 calibration strategy onto the query library fragment ion *m/z* values across the entire mass range (Supplementary Fig. 4). This calibration masks inherent issues with the library sufficiently enough to appear that the analysis is performing well. However, DIALib-QC is able to identify the fragment ion mass error prior to DIA analysis, which produced a nearly 30% increase in the number of peptide identifications.

Further, seven sample-specific libraries were created (see Methods) and analyzed with DIALib-QC to highlight their differences juxtaposed with their effects on DIA analysis. The number of peptides and protein groups identified with PeakView or Spectronaut was consistent regardless of which library was used in the DIA analysis, except with the q3bad library (Fig. 2b), but the distinct differences in the libraries are evident from the DIALib-QC results (Supplementary Data 2). DIALib-QC identified the small fraction (0.1%) of decoy assays in the base library, that were left over from the DDA analysis during the library generation process. These assays were therefore removed in the conflict and good libraries and thus decoy numbers were confirmed as zero by DIALib-QC (Supplementary Data 2). However, in silico decoy assays are often appended to the library in tools such as OpenSWATH for false discovery rate (FDR) estimation. For DIA tools (such as Spectronaut and PeakView) that apply their own mechanism to estimate the FDR, in silico decoy assays can be added to the library to perform an entrapment analysis^13^. During entrapment analysis, the false positive identifications of decoy peptides are estimated by matching to these decoy assays following DIA analysis, which can be used to calculate a realized FDR that is compared to the FDR estimate provided by the software. In either case, knowledge of the number of decoy assays is essential to determine if sufficient and not excessive assays are available to perform adequate DIA analysis.

**Fig. 3:**
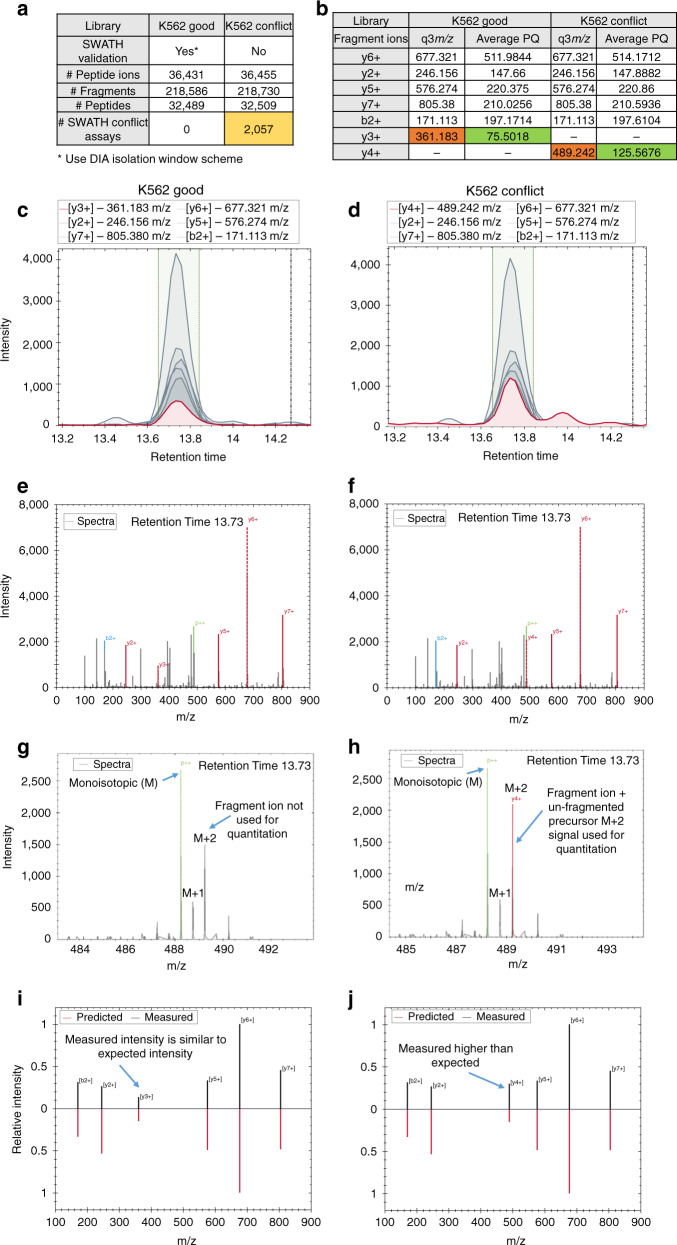
Effect of conflict assay on the peptide quantitation in Spectronaut analysis. **a** DIALib-QC assessment reports the number of DIA conflict assays in both libraries. **b** Comparison of average peak quantities (PQ) of peptide ion GLQTSQDAR.2 between the two libraries (highlighted in orange and green) for the most intense six fragment ions observed in all five DIA/SWATH replicates. **c**, **d** Quantitative MS2 analysis based on extracted ion chromatograms (XICs) of peptide GLQTSQDAR.2 with both libraries eluted at the same RT. **e**, **f** MS2 spectrum of peptide GLQTSQDAR.2 in both libraries. p++ denotes un-fragmented precursor ion with [M + 2H]^2+^ charge. **g**, **h** Zoomed MS2 spectrum highlighting the overlap of un-fragmented precursor isotopic envelope (M, M + 1, M + 2) with y4+ ion in conflict library. **i**, **j** Correlation plots between measured (black) and predicted (red) relative fragment y4+ ion intensity showed higher similarity in the good than in the conflict library.

Next, in the conflict library, DIALib-QC found 2057 peptide assays to have q3 fragment ions that fall in the same ion bin as the parent q1 (Fig. 3a). To demonstrate the effect of these conflict assays on the peptide quantitation, we compared the average peak quantities (PQ) between the two libraries (good and conflict) for the most intense 6 fragment ions of peptide ion GLQTSQDAR.2 in Spectronaut analysis (Fig. 3b). Both libraries showed similar contribution of five fragment ions in peptide quantitation whereas the sixth fragment ion, y4+ (489.24 *m/z*) in conflict contributes more than y3+ (361.18 *m/z*) in the good library (colored red to the total PQ) (Fig. 3c, d). The overestimating of peptide quantitation was mainly attributed by the residual precursor signal (denoted as p++) in MS2 spectrum (Fig. 3e, f). In the conflict library, the un-fragmented precursor [M + 2H]^2+^ ion is overlapped with the y4+ ion (489.242 *m/z*) used for quantitation of the peptide, whereas in the good library, the y4+ ion was not used as it was removed during DIA verification (Fig. 3g, h). The correlation plots of relative intensities of fragment ions clearly indicates that y3+ ion in good library showed higher similarity in measured (black) and predicted (red) intensities whereas y4+ ion in conflict library was measured higher than expected intensity (Fig. 3i, j). This observation further supports that these conflict assays can affect peptide quantitation when overlapping residual precursor signal is measured with the q3 fragment ion intensity (Supplementary Fig. 5). Although the conflict library had little effect on peptide identification, DIALib-QC identified assays that can affect peptide quantitation.

Further, comparing the good to the raw library, DIALib-QC highlights similar numbers of peptide and fragment ions but the raw library contains ~7 times more average fragments per peptide ions (Supplementary Data 2). Limiting redundant spectra (raw library) to the top six fragments per peptide ion in the DIA tools, both libraries gave similar identifications (Fig. 2b). This indicates that the score distribution of six co-eluting fragment ions in both libraries were similar and do not affect the sensitivity of the DIA analysis (Supplementary Fig. 6). Lastly in the q3bad library, DIALib-QC flagged the average mass difference of 0.0064 Dalton in fragment ion *m/z* values (Supplementary Data 2), that results into a decrease in identifications of peptides (18%) and protein groups (8%) with PeakView (Fig. 2b), consistent with the PHL libraries evaluations. The effect of fragment ion mass errors on the score distribution in PeakView is exemplified with peptide VAPDEHPILLTEAPLNPK (Supplementary Fig. 7). To demonstrate the use of DIALib-QC in constructing an error-free library, it’s repairing option was applied on the q3bad library. This option recomputes the theoretical *m/z* values of the fragment ions, generating a mass corrected library (q3bad_mz_corrected) (Supplementary Data 2). In this corrected library, DIALib-QC reported no average mass difference in the fragment ions but identified five conflict assays (Supplementary Data 2). These assays became conflict assays after the mass correction step as they now fall in the SWATH precursor window and were filtered sequentially by running the DIALib-QC filter conflict assay option. Two resultant final libraries were exported, one consisting of all conflict assays for inspection, and the other consisting of error-free assays (q3bad_mz_corrected_clean library) for use in DIA analysis. Additionally, characteristics of the error-free library (q3bad_mz_corrected_clean) are graphically represented in Supplementary Fig. 8. DIALib-QC assessment of the error-free library showed no problem assays (Supplementary Data 2) and hence mimics its good counterpart, as an ideal spectral library for the downstream DIA/SWATH analysis.

To assist in defining high confidence DIA analysis, we introduce DIALib-QC, describe its features, and highlight its capabilities in successfully assessing and improving the quality of spectral libraries for DIA analysis. We demonstrate the effect of library correctness attributes in both public and in-house spectral libraries on the performance of DIA analysis along with an option to obtain a clean ion library without mass errors and conflict assays. We recommend assessing and correcting spectral ion libraries with DIALib-QC, prior to DIA analysis, to ensure correct identification and accurate quantitation of peptides and proteins from DIA data processing tools.

## Methods

### K562 cell culture and protein digestion

K562 cells (ATCC CCL-243, human bone marrow myeloid leukemia cell line) were cultured at 37 °C with 5% CO_2_ in EMEM (ATCC 30-2003) supplemented with 10% fetal bovine serum. Cells were grown to 70% confluence, harvested, lysed, and proteins denatured in 8 M urea, 0.1% RapiGest, and 100 mM NH_4_HCO_3_. Protein content was determined by bicinchoninic acid assay (Thermo-Fisher). Proteins were reduced with 5 mM TCEP (60 min, 37 °C), alkylated with 10 mM iodoacetamide (30 min, room temperature, and darkness), digested using 1:50 trypsin (enzyme/protein, Promega), and samples desalted with tC18 SepPak cartridges (Waters).

### Liquid chromatography–mass spectrometry

Peptides were separated with a NanoLC 400 interfacePlus HPLC system (Eksigent) configured in micro-flow mode and emitted into a TripleTOF 6600 mass spectrometer (SCIEX). Peptides were trapped on a 10 × 0.3 mm trap cartridge Chrom XP C18CL, 5 µm, 120 Å (Eksigent) at 10 µL/min, and separated on a ReproSil-Pur C18-AQ, 2.4 µm, 150 × 0.2 mm (Dr. Maisch GmbH) at 5 µL/min using a gradient from 3 to 35% B in 98 min, 35 to 40% in 5 min, 40 to 80% B in 2 min, isocratic flow at 80% B for 8 min, 80 to 3% B in 2 min, and isocratic at 3% B for 25 min. Data were acquired using data-dependent acquisition (DDA) and data-independent acquisition (DIA) mode. For both DDA and DIA acquisitions with micro-flow chromatography, the ion source was equipped with a 25 µm Turbo Ion Spray probe (SCIEX) and parameters were as follows: ISVF = 5500, GS1 = 15, GS2 = 15, CUR = 25, and TEM = 100.

### DDA mass spectrometry for spectral library generation

Two DDA replicates of each of top-10, top-20, top-40, top-50, and top-100 mode configuration were acquired in the TripleTOF 6600 system while adjusting the accumulation time for MS1 and MS2 scans and keeping a constant total cycle time of 2.8 seconds (s) for each run. Precursor spectra (400–1250 *m/z*) and fragment ion spectra (100–1500 *m/z*) were collected with dynamic accumulation. The selected precursors were then added to a dynamic exclusion list of 20 s. Rolling collision energy with a collision energy spread of 15 eV was used for fragmentation to mimic SWATH like fragmentation conditions.

### DIA/SWATH mass spectrometry

DIA/SWATH data were collected with an MS/MS^ALL^ SWATH^TM^ Acquisition method using 100 variable acquisition windows^14^, each with a 1 Da overlap with the previous window. SWATH-MS2 spectra were collected in high-sensitivity mode from 100 to 1500 *m/z* with 25 milliseconds (ms) accumulation time. Before each SWATH-MS cycle, an MS1 survey scan in high-resolution mode was recorded with a 250 ms accumulation time, resulting in a total duty cycle of ~2.8 s. Five analytical replicates of the K562 cell digest were measured for statistical confidence.

### Spectral and assay library generation

All DDA wiff files were converted to profile mzML files using the ABSciex MS Data Converter version 1.3 beta. The Trans-Proteomic Pipeline (TPP)^15^ (5.2.0) was used for the analysis of the shotgun proteomics runs. The datasets were searched with Comet^16^ (2017.01r1) against the full nonredundant, canonical human proteome from UniProtKB/Swiss-Prot (2018_07) with 20,270 ORFs and appended contaminants, shuffled decoy sequences and iRT peptides (Biognosys). Carbamidomethyl (57.0214 Da) modification on cysteines was used as a fixed modification; oxidation (15.9949 Da) on methionine as a variable modification. Parent mass error was set to ±50 ppm., fragment bin tolerance was set to 0.05 *m/z*. The search identifications were combined and statistically scored using PeptideProphet^17^ and iProphet^18^ within the TPP^15^. MAYU^19^ (1.07) was used to select an iProphet cutoff of 0.78, resulting in a protein FDR of 1% (Supplementary Data 3). SpectraST^20^ was used in library generation mode with CID-QTOF settings and a consensus library was consecutively generated according to Rosenberger et al.^21^ using spectrast2tsv.py (msproteomicstools 0.2.2; https://pypi.Python.org/pypi/msproteomicstools).

### Modifications of assay libraries

We generated two different sets of spectral ion libraries (eleven libraries in total) to demonstrate the application of DIALib-QC. The first set is based on the PHL library by Rosenberger et al.^21^ (available at www.swathatlas.org) and the second set is based on an in-house generated sample-specific library from K562 cells. To demonstrate DIALib-QC, all libraries were modified to ensure consistency in RT and assay coordinates including the number of precursor (q1), and fragment ions (q3) per precursor, both in their respective charge states, and relative fragment ions intensities. The DIALib-QC assessment reports of all libraries are provided in Supplementary Data 2.

The first set of libraries, based on the PHL ion library, was computationally modified by adding a q3 delta *m/z* of 0.01, 0.02, and 0.05 Daltons to the theoretical q3 *m/z*. A total of four PHL libraries named good (control), q3bad_0.01, q3bad_0.02, and q3bad_0.05 were generated. The good library uses theoretical q3 *m/z* values (monoisotopic masses) and is compared to the other three perturbed libraries. The purpose behind perturbing computationally the q3 *m/z* values is to determine the effect of q3 mass errors on the sensitivity of identification and relative quantitation in DIA experiments, here using both PeakView and Spectronaut. Such *m/z* deviations from theoretical values can occur while e.g., generating libraries from observed fragment ion masses instead of theoretical ion masses, and the degree to which mass errors occur can reflect differences in the quality of the data used to generate these libraries.

The second set of libraries are in-house sample-specific spectral/DIA libraries generated from ten replicates of a K562 cell digest acquired in DDA mode, using the TPP^15^ pipeline and SpectraST^20^ workflow. Since DIA ion library generation is a multiple step process, an unoptimized pipeline can result in low quality and erroneous spectral ion libraries. The purpose of generating these sets of libraries is to mimic the error prone library generation steps, as highlighted by DIALib-QC in its diagnostic criteria of the assessment report (Supplementary Data 1). This information is used to show the effect of bias in the ratio of target/decoy assays in a library (base library), mass accuracy (theoretical vs. observed q3 mass values), redundant spectra in raw library, RT fit (correlation of [M + 2H]^2+^ and [M + 3H]^3+^ of same peptide ion) and conflict assays (conflict library) on to the performance of DIA experiments. To do so, the in-house libraries were implicitly perturbed at the SpectraST step, resulting in seven, different libraries described below.

*Library 1: K562base* (*Consensus spectra* + *theoretical fragment ion m/z values* + *decoy assays* + *conflict assays*): The base library was generated from consensus spectra using theoretical q3 mass values in SpectraST. SpectraST records all theoretical fragment ion *m/z* values and keeps them with the −e option enabled in the spectral library. The base library includes both target and decoy assays. Decoy assays are assays to peptides that do not exist in the human (target) proteome and are inherited from the DDA analysis during the library generation step. Peptide sequences identified from these decoy assays (referred to as decoy peptides in DIA analysis) are therefore known to be false. Further, the library was limited to the top 6 fragments per precursor without enabling the SWATH verification which kept the conflict assays in the library.

*Library 2: K562 conflict (Consensus spectra* + *theoretical fragment ion m/z values* + *conflict assays*): This library was generated by removing the decoy assays from Library 1. Library 2 contains conflict assays (assays which are not verified with the SWATH definition file). This SWATH definition file contains precursor isolation windows with the information of mass range and width used in SWATH mode. The objective to construct this library was to understand how fragment ions that fall in the parent ion bin(s) impact the identification and quantitation of a peptide using both Spectronaut and PeakView analysis. The DIALib-QC tool reports the total number of precursor and fragment ions *m/z* values that contribute to conflict assays in the library.

*Library 3: K562 good (consensus spectra* + *theoretical fragment ion m/z values*): This library was generated from consensus spectra using theoretical fragment ion *m/z* values in SpectraST. K562 good was used as control library and was compared to other perturbed libraries. Library 3 was verified with the DIA/SWATH definition file used for sample acquisition and which ensures that fragments that fall in the parent ion swath bin(s) will not be selected.

*Library 4: K562 q3bad (consensus spectra* + *observed fragment ion m/z values*): This library was generated from consensus spectra in the SpectraST pipeline without enabling the -e option, which resulted in storing the observed fragment ion *m/z* values in the spectral library.

*Library 5: K562 raw (redundant spectra* + *theoretical fragment ion m/z values*): The raw spectral library was generated directly from redundant spectra and contains non-unique entries resulting from multiple observations of the same peptide ion. All fragment ions of the same peptide ion found in all sample replicates above an iProphet probability threshold were used to generate the spectral library. The K562 raw spectral library uses theoretical fragment ion *m/z* values.

*Library 6: K562 q3bad_mz_corrected (consensus spectra* + *corrected theoretical fragment ion m/z values)*: This library was generated from Library 4 (q3bad) by correcting the observed to theoretical fragment ion *m/z* values.

*Library 7: K562 q3bad_mz_corrected_clean (consensus spectra* + *corrected theoretical fragment ion m/z values* + *removed conflict assays*): This library was generated from Library 6 by removing the conflict assays identified in reference to the SWATH definition file by using the filter conflict assays option of DIALib-QC. The resultant library is free from any mass errors and conflict assays.

Further, for each spectral library (in both sets), two different library formats were used for analysis based on the DIA software, PeakView or Spectronaut. For each library, PKV.txt formatted libraries were generated using the SpectraST workflow. In the PKV format used in PeakView (SWATH 2.0) analysis, iRT values of all assays were shifted to positive values by adding −60.6 and 51.6 for PHL and K562 set of libraries respectively to prevent the failure in importing the complete information of the library. For Spectronaut analysis, negative iRT values are permissible in PKV inputs and did not require additional manipulation. DIALib-QC provides assessment reports for all libraries (DDA derived) in both library formats, PeakView and Spectronaut (Supplementary Data 2).

In addition, DIALib-QC is fully capable to evaluate DIA derived libraries constructed using untargeted workflows in Spectronaut and DIA-Umpire^22^. The tool supports Spectronaut format libraries regardless of, if it is built from DDA or DIA data. Similarly, DIA-Umpire analysis can be used to generate a library directly from DIA data using the SpectraST pipeline, which produces library formats (OpenSWATH and PeakView) compatible with DIALib-QC. In case of other formats, users can transform their library to formats compatible with DIALib-QC for its assessment and repair.

### DIA/SWATH data analysis

Two different DIA software tools were used in this study, Sciex PeakView and Biognosys Spectronaut, to perform the targeted data extraction of five analytical DIA replicates.

### PeakView (SWATH 2.0)

PeakView.tsv assay libraries generated as described above were appended with iRT peptide assays (protein labeled as [RT-Cal protein]). The RT calibration for all peptides in the ion library was performed based on the iRT peptide elution profiles for an adjusted RT window at a 75 ppm XIC extraction width with PeakView version 2.2 and the SWATH 2.0 plug-in MS/MS(ALL) using SWATH App module (v2.0) (SCIEX). To import all peptides from the library, the number of peptides per protein was set to the maximum value of 9999 with 6 transitions per peptide. The FDR threshold was set at 1%, excluding both modified and shared peptides. The processed results were exported to Microsoft Excel files (Supplementary Data 4).

### Spectronaut

For Spectronaut, the assay libraries were used directly as generated and described above. The HTRMS converter was used to convert the raw WIFF files into HTRMS files which were imported into Spectronaut version 13.3.190726.43655 (Laika), (Biognosys, Switzerland). For the nonlinear iRT calibration strategy, a dynamic window was used for both mass tolerance and XIC RT extraction window. Preprocessing of MS1 and MS2 calibration strategy was enabled. The decoy assays were dynamically generated using the scrambled decoy method with a set size of 1 as a fraction of the input library size. The identification was performed using the normal distribution estimator with precursor and protein identification results filtered with a *q* value of <0.01. For quantification, MS2 ion peak areas of quantified peptides were summed to estimate the protein peak areas (Supplementary Data 5).

### Reporting summary

Further information on research design is available in the Nature Research Reporting Summary linked to this article.

## Supplementary information

Supplementary Information

Peer Review File

Description of Additional Supplementary Files

Supplementary Data 1

Supplementary Data 2

Supplementary Data 3

Supplementary Data 4

Supplementary Data 5

Reporting Summary

## Data Availability

Data that support the findings of this study are available via ProteomeXchange with identifier PXD020953 via the PRIDE^23^. Any other relevant data are available from the corresponding author on request.

## Code availability

The user-friendly web version of the DIALib-QC tool is available at https://db.systemsbiology.net/sbeams/cgi/PeptideAtlas/AssessDIALibrary. DIALib-QC code, installation guide and instructions on how to run the full pipeline including repairing the spectral library can be found at http://www.swathatlas.org/DIALibQC.php. DIALib-QC v1.0 is also freely available at https://github.com/CharuKMidha/DIALib-QC under a GNU licence.
