# Supplementary material for: DIALib-QC an assessment tool for spectral libraries in data-independent acquisition proteomics
Source: Nat Commun. 2020 Oct 16;11:5251. doi: 10.1038/s41467-020-18901-y (PMC7567827; doi:10.1038/s41467-020-18901-y)
Supplement: Supplementary file 1 — Supplementary Information [file 41467_2020_18901_MOESM1_ESM.pdf]

Supplementary Information

DIALib-QC an assessment tool for spectral libraries in data-independent acquisition proteomics

Mukul K. Midha, David S. Campbell, Charu Kapil, Ulrike Kusebauch, Michael R. Hoopmann, Samuel L. Bader, and Robert L. Moritz*

*Address correspondence to: Robert L. Moritz, Institute for Systems Biology, 401 Terry Ave N, Seattle, WA 98109, USA, Email: rmoritz@systemsbiology.org

Contents

Supplementary Figure 1. Workflow of K562 library generation and PHL modification. 3

Supplementary Figure 2. Correlation fit between the retention time (RT) of $[M+2H]^{2+}$ and $[M+3H]^{3+}$ charge states of common peptides. 5

Supplementary Figure 3. Score distribution of Targets and Decoys of PHL sets of libraries with PeakView analysis. 6

Supplementary Figure 4. Spectronaut pre-processing MS2 calibration strategy with PHL libraries analysis... 7

Supplementary Figure 5. Effect of conflict assays on the quantitation of peptide ion GLQTSQDAR.2 in PeakView analysis. 8

Supplementary Figure 6. Score distribution of targets and decoys of the K562 set of libraries with PeakView analysis. 10

Supplementary Figure 7. Effect of q3 mass error in spectral libraries on the score distribution of peptide ion VAPDEHPILLTEAPLNPK.3 in PeakView analysis. 11

Supplementary Figure 8. Graphical representation of characteristics of the spectral library ....

by DIALib-QC. 13

Supplementary Figures

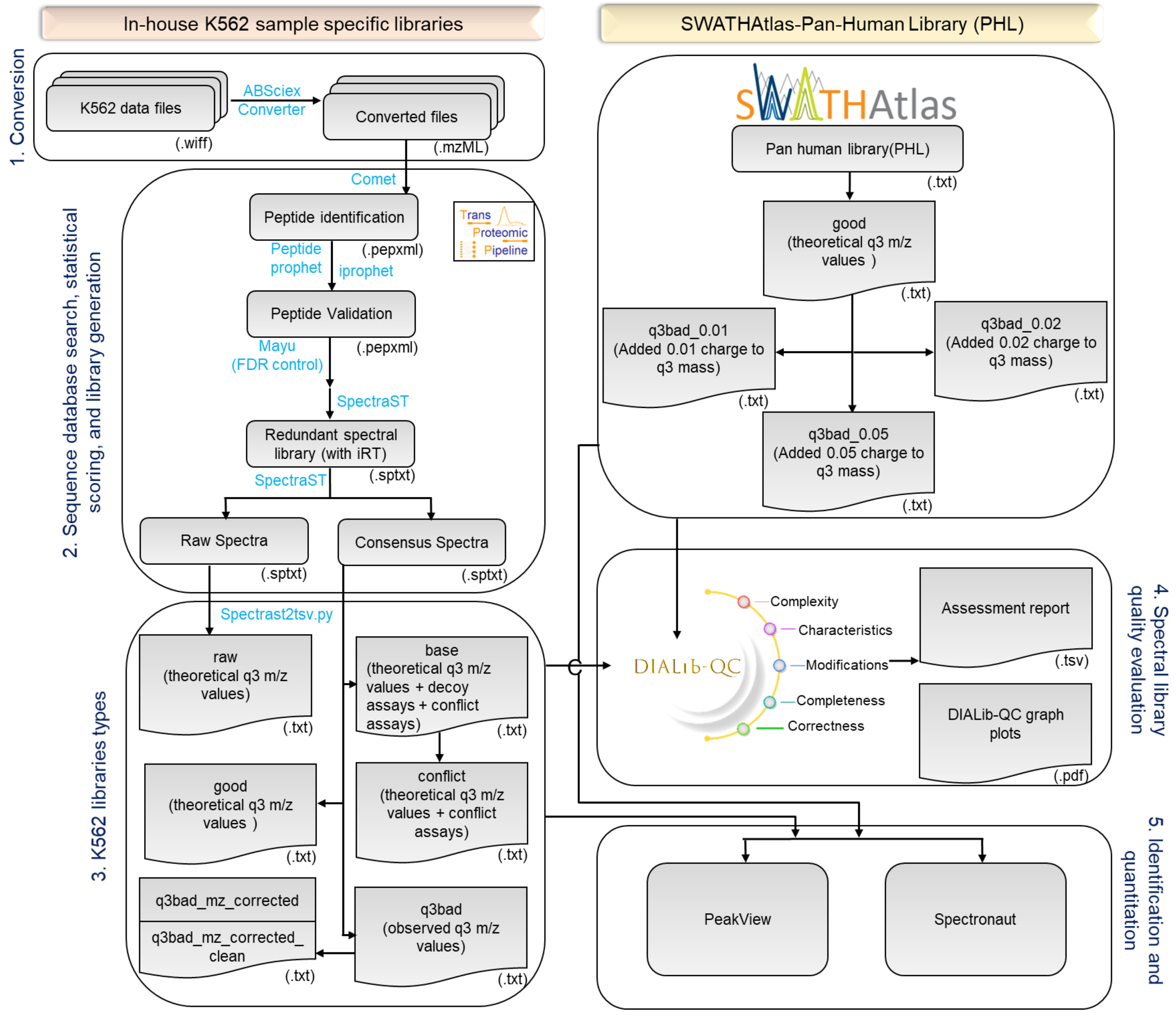

Supplementary Figure 1. Workflow of K562 library generation and PHL modification.

For the K562 in-house sample specific library, the workflow starts with measuring the samples and converting the data files into mzML format using the ABSCIEX converter **(part 1)**. Next, the mzML files are searched against a protein sequence database to identify the peptide sequences

using the Comet search engine. The identified sequences are then validated statistically using PeptideProphet and iProphet. MAYU is applied to control the FDR at the protein level. Using SpectraST, confidently assigned spectra are converted into a redundant spectral library and retention times are normalized using iRT reference peptides, then a consensus spectrum library is generated **(part 2)**. The spectrast2tsv.py script generates an ion library from the consensus library. The raw ion library is extracted directly from the redundant spectra library and six other consensus ion libraries were made for comparison **(part 3)**. All ion libraries are evaluated with the DIA Library Quality Control (DIALib-QC) tool and their assessment reports are generated **(part 4)**. Finally, the performance of the libraries is compared based on the identification and quantitation outputs from the PeakView and Spectronaut analysis **(part 5)**. The public Pan human library (PHL) was downloaded from SWATHAtlas.org and further modified to understand the effect of inaccurate libraries on DIA analysis. We removed decoy assays from the original PHL library to generate the good library and further modified the q3 *m/z* values to generate three modified libraries. The PHL derived libraries are also evaluated with DIALib-QC, and the incorrect q3 *m/z* values are summarized in the assessment reports.

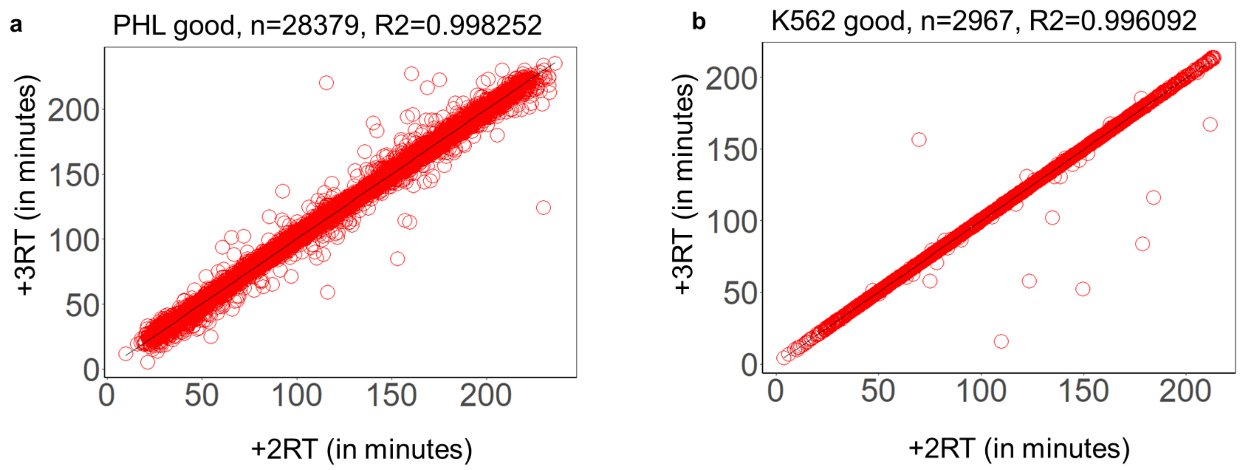

Supplementary Figure 2. Correlation fit between the retention time (RT) of $[M+2H]^{2+}$ and $[M+3H]^{3+}$ charge states of common peptides.

a) PHL good, **b)** K562 good. N refers to the number of peptide pairs with $[M+2H]^{2+}$ and $[M+3H]^{3+}$ charge states. For both libraries, peptide pairs demonstrate similar chromatographic behavior and correct RT normalization using reference peptides as indicated by high positive retention time correlation of >0.99. The x and y axis for both libraries represents the $[M+2H]^{2+}$ and $[M+3H]^{3+}$ charge state of a common peptide respectively.

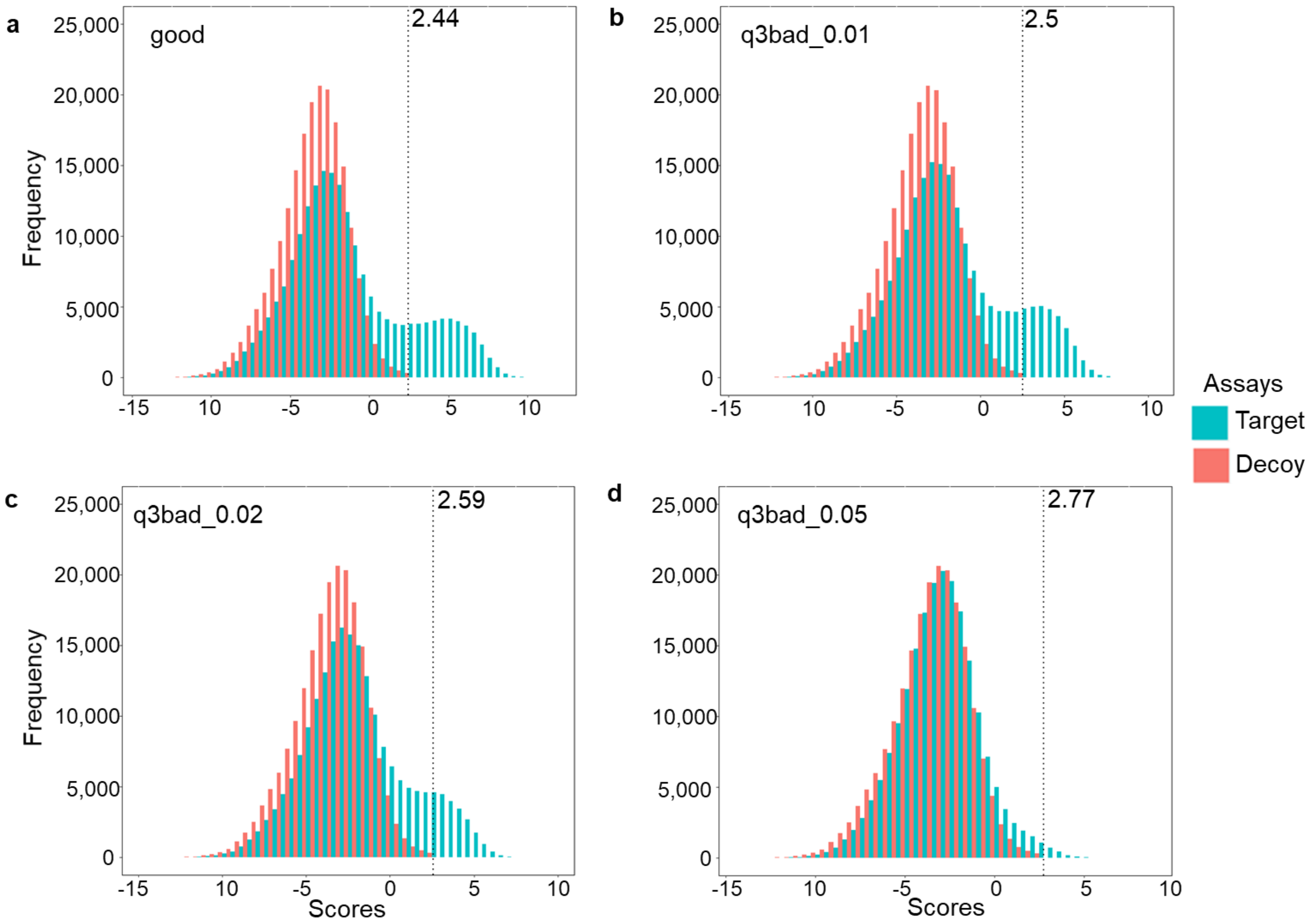

Supplementary Figure 3. Score distribution of Targets and Decoys of PHL sets of libraries with PeakView analysis.

a) good, **b)** q3bad_0.01, **c)** q3bad_0.02, **d)** q3bad_0.05. For both target and decoy peak groups, PeakView computes scores based on several chromatography and spectral attributes, and FDR estimation is based on the distributions of target and decoy peptides. The increase in the q3 delta mass (mass difference between reported and theoretical q3 *m/z*), affects the peak group score distribution of target assays (cyan colored), cut-off threshold by 1% FDR filtering (dotted line). The number of target assay identifications decreases with the increase in the q3 delta mass based on the score distribution of target and decoy assays (red colored).

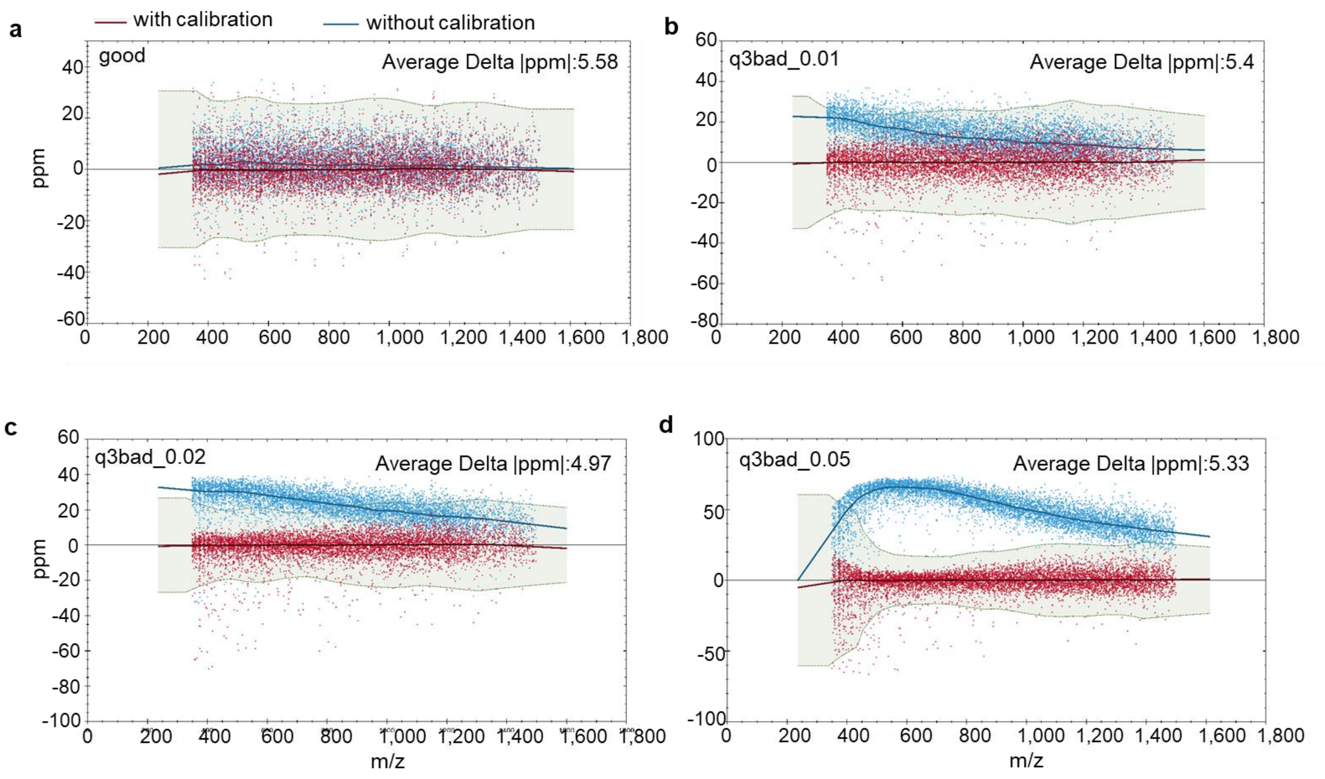

Supplementary Figure 4. Spectronaut pre-processing MS2 calibration strategy with PHL libraries analysis.

a) good, **b)** q3bad_0.01, **c)** q3bad_0.02, **d)** q3bad_0.05. Spectronaut estimates the q3 mass error (colored blue) and calibrates the library q3 *m/z* values to fit with the DIA data q3 *m/z* values (colored red) across the entire mass range. This becomes evident with the PHL set of libraries analyzing the same DIA/SWATH file. As q3 delta mass is increased, Spectronaut brings the delta mass near to zero by adjusting the libraries *m/z* values. Spectronaut failed for the q3bad_0.05 library and was unable to adjust all library *m/z* values above a certain threshold (here 100 ppm), especially in the lower mass region of 400 to 600 *m/z* resulting in some loss of peptide and protein identifications.

a

Library	K562 good	K562 conflict
SWATH validation	Yes*	No
# Peptide ions	36,431	36,455
# Fragments	218,586	218,730
# Peptides	32,489	32,509
# SWATH conflict assays	0	2,057

* use DIA isolation window scheme

b

Library	K562 good		K562 conflict	
Fragment ions	q3*m/z*	Average PQ	q3*m/z*	Average PQ
y6+	677.321	236270.1	677.321	236270.1
y2+	246.156	50032.69	246.156	50032.69
y5+	576.274	95560.6	576.274	95560.6
y7+	805.38	103113.2	805.38	103113.2
b2+	171.113	60600.09	171.113	60600.09
y3+	361.183	28191.18	-	-
y4+	-	-	489.242	56363.05

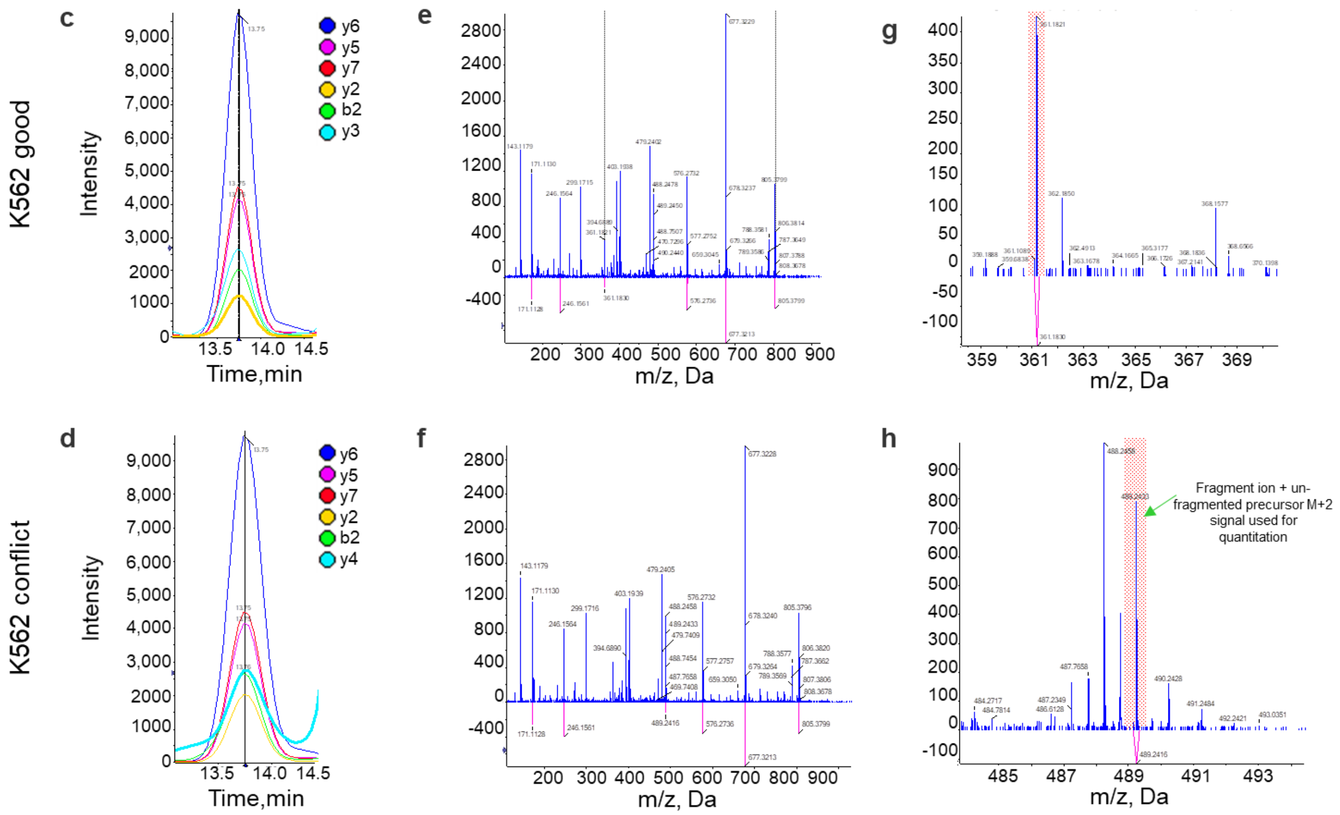

Supplementary Figure 5. Effect of conflict assays on the quantitation of peptide ion GLQTSQDAR.2 in PeakView analysis.

a) DIALib-QC assessment reports the number of DIA conflict assays by comparing the good with the conflict library. **b)** Comparison of average peak quantities (PQ) between good and conflict library, estimated by PeakView for the top-6 fragment ions observed in all five DIA replicates. Interestingly, PeakView estimates identical peak areas of five fragments in both libraries with no change in peak quantities. Different fragment ions between the libraries are highlighted with

orange colored cells and their peak quantities are colored in green. **c)** and **d)** Quantitative MS2 analysis based on extracted ion chromatograms (XICs) of peptide GLQTSQDAR.2 with the good and the conflict library, eluted at the same retention time. It shows similar contributions of five fragment ions in both libraries except the contribution of the y3+ (361.183 *m/z*) ion in the good library and the y4+ (489.242 *m/z*) ion in the conflict library to the same peptide (highlighted in turquoise color to the total peak quantities). **e)** and **f)** The plot provides correlation of expected and measured relative fragment ion intensities for peptide GLQTSQDAR.2. **g)** and **h)** Zoomed version of MS2 spectrum highlighting unfragmented precursor isotopic envelope (M, M+1, M+2). The M+2 ion is overlapped by y4+ (489.242 *m/z*) which is used for quantitation of peptide GLQTSQDAR.2 in using the conflict library. In the good library, y4+ (489.242 *m/z*) was not used for quantitation as it was removed upon DIA verification, instead the y3+ (361.183 *m/z*) was used.

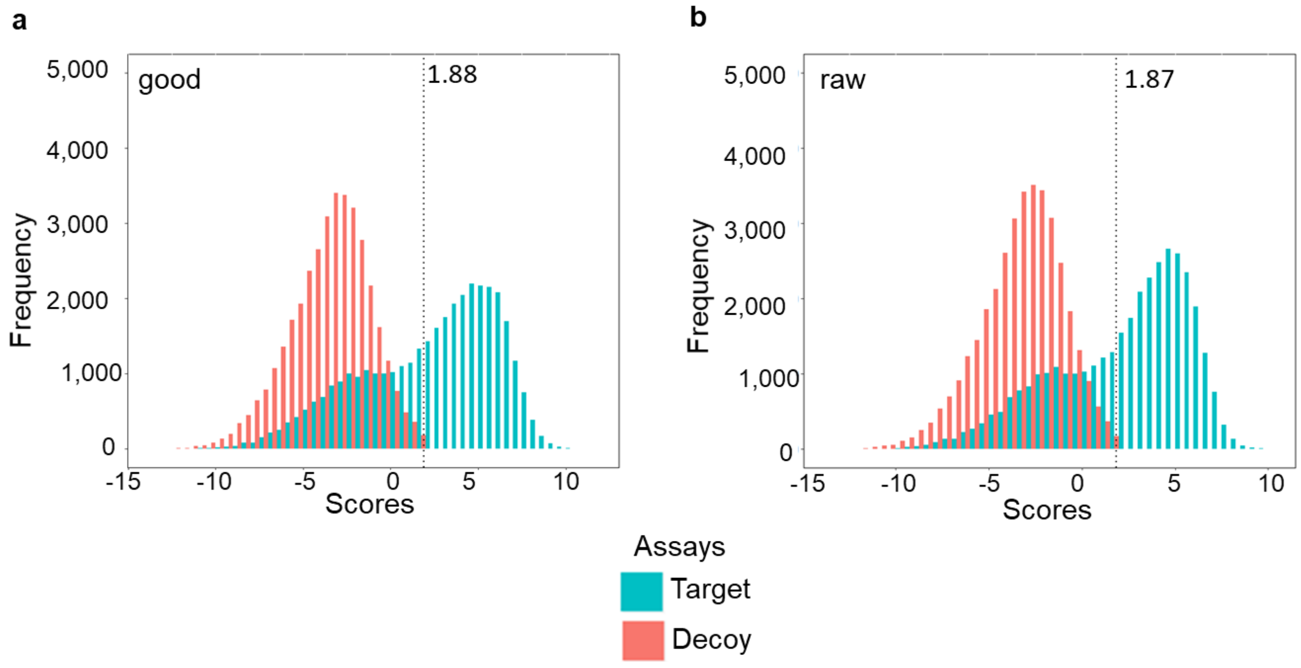

Supplementary Figure 6. Score distribution of targets and decoys of the K562 set of libraries with PeakView analysis.

a) K562 good, and **b)** K562 raw. The score distribution of both targets (cyan colored) and decoys (red colored) and FDR intercept values (dotted lines) estimated by PeakView are similar for both libraries, indicating similar identifications of peak groups.

a

Library	K562 good	K562 q3bad
SpectraST	with “-e” *	without “-e”
Format	PeakView	PeakView
# Pepions	36452	36452
# Fragments	218712	218712
# Peptides	32503	32503
# precursor_ok	36452	36452
# precursor_bad	0	0
# fragment_ok	216942	9947
# fragment_bad	0	206995
fragment_ave_mdiff	0	0.0064

* “-e” use theoretical mass

b

Library	K562 good	K562 q3bad
Fragment ions	q3 *m/z*	q3 *m/z*
b2+	171.1128	171.1090
y6+	639.3824	639.3783
y5+	568.3453	568.3393
y7+	768.425	768.4402
y2+	244.1656	244.1641
y8+	869.4727	869.4727

c

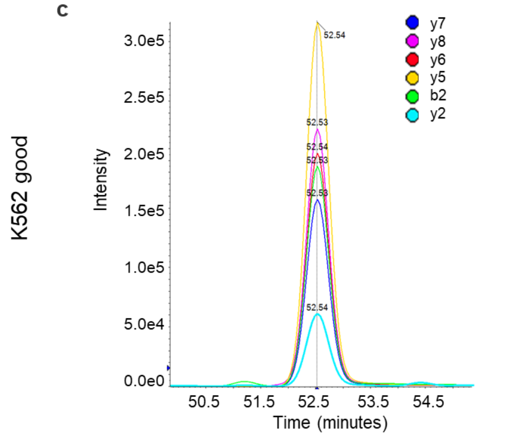

e

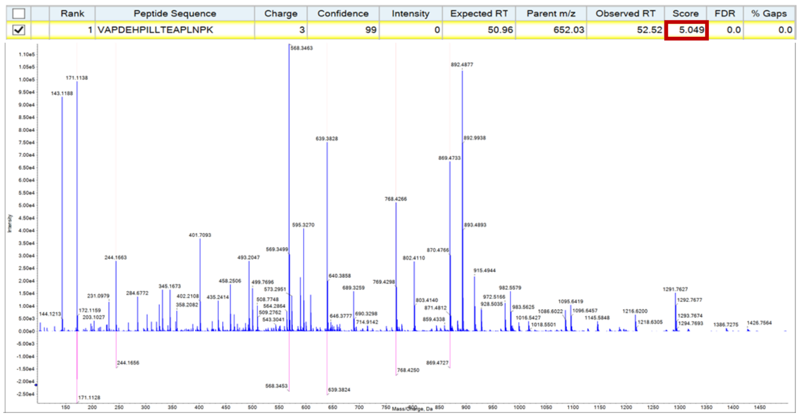

d

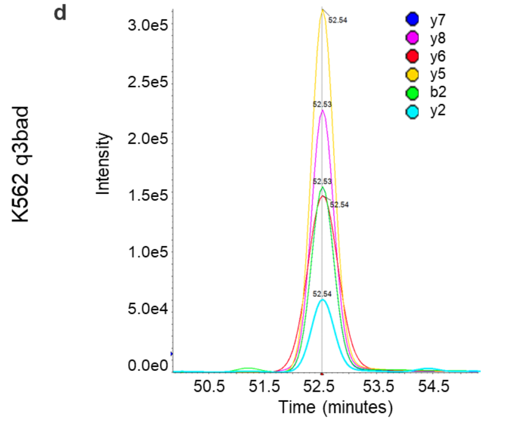

f

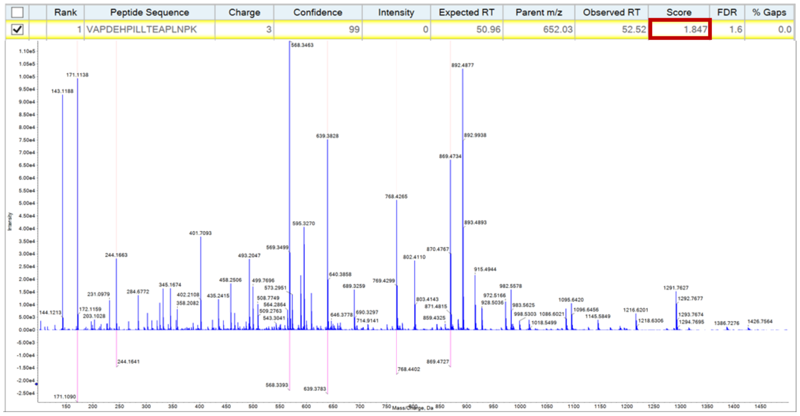

Supplementary Figure 7. Effect of q3 mass error in spectral libraries on the score distribution of peptide ion VAPDEHPILLTEAPLNPK.3 in PeakView analysis.

Comparison of score that accounts for accurate mass of MS2 scans of single peptide ion VAPDEHPILLTEAPLNPK.3 estimated in good with q3bad library in PeakView analysis.

a) DIALib-QC assessment report highlighting the number of bad fragment ions (#q3 bad) and delta q3 mass error (yellow colored) by comparing the good with q3bad library. **b)** Comparison of top 6 q3 fragment ions *m/z* and type in the good and q3bad library. **c)** and **d)** Extracted Ion Chromatogram (XIC) of VAPDEHPILLTEAPLNPK.3 peptide with the good and q3bad library eluted at the same retention time, highlighting the same peptide abundances estimated by PeakView. **e)** and **f)** PeakView estimates the score of VAPDEHPILLTEAPLNPK.3 is 5.049 (highlighted in red box) with less than 1% FDR using the good library while the score of the same peptide ion is 1.847 (highlighted in red box) with more than 1% FDR value using the q3bad library. This illustrates that PeakView considers the *m/z* error attribute in the spectral library for peak group scoring.

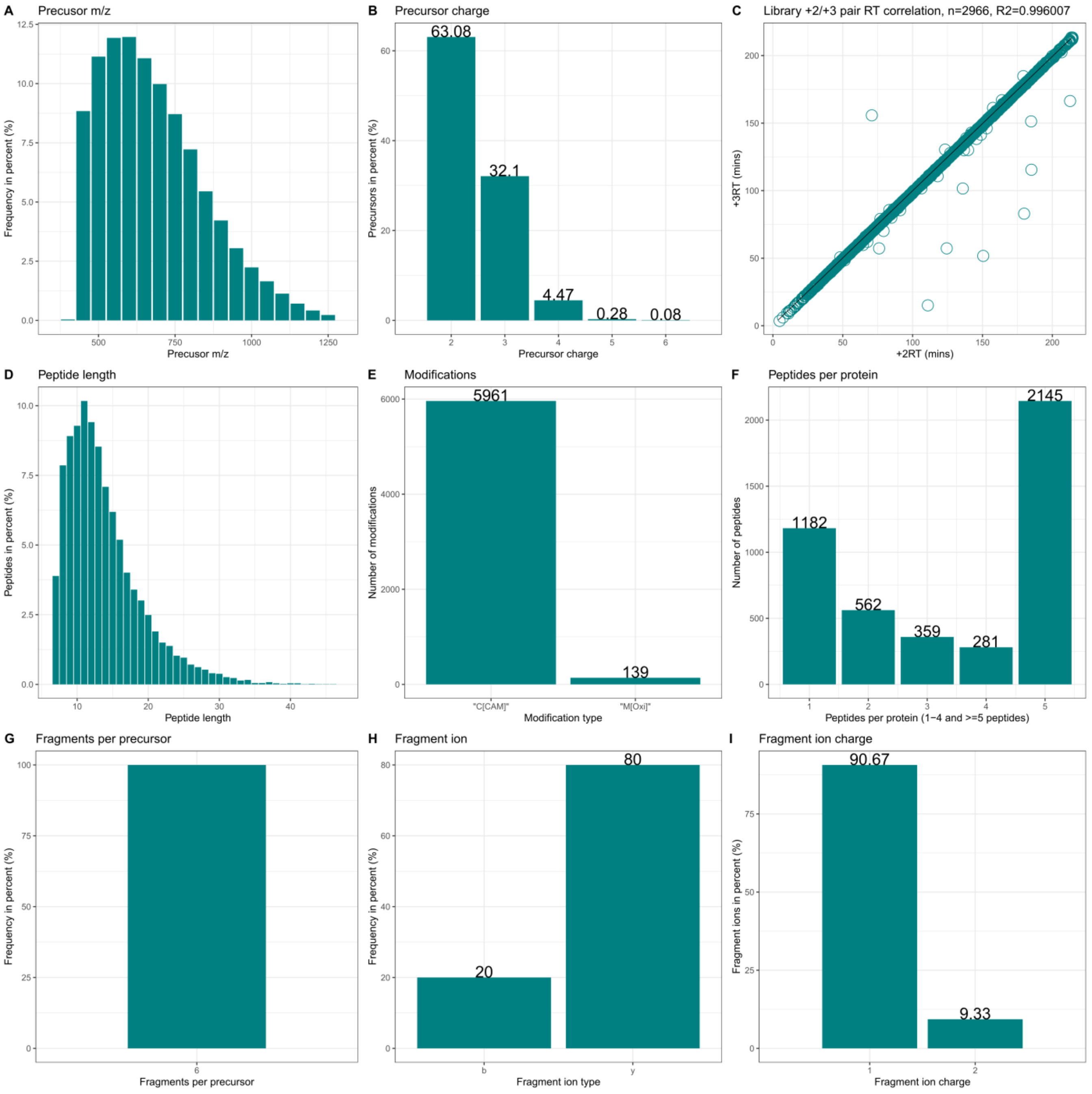

Supplementary Figure 8. Graphical representation of characteristics of the spectral library by DIALib-QC.

A) Distribution of precursor *m/z* values across the acquired mass range in the ion library. **B)** Frequency of precursor charge states observed in the ion library. **C)** Correlation of retention time (RT) fit of +2 and +3 charge states of the same peptide in the ion library estimated by DIALib-QC to assess the quality of the library. N refers to the number of peptide pairs with +2 and +3 charge states. **D)** Distribution of peptide length in the ion library. **E)** Frequency and type of peptide modification observed in the assay library. CAM: carbamidomethylation, Oxi: oxidation. **F)** The graph depicts the number of K562 (human) peptides per protein in the library. **G)** Distribution of the number of fragment ions per precursor. **H)** Frequency of observed b- and y- ion fragments with CID fragmentation in the ion library. **I)** Frequency of fragment ion charge states observed in the ion library.
