# Supplementary material for: DIALib-QC an assessment tool for spectral libraries in data-independent acquisition proteomics
Source: Nat Commun. 2020 Oct 16;11:5251. doi: 10.1038/s41467-020-18901-y (PMC7567827; doi:10.1038/s41467-020-18901-y)
Supplement: Supplementary file 3 — Description of Additional Supplementary Files [file 41467_2020_18901_MOESM3_ESM.pdf]

Description of Additional Supplementary Files

Supplementary Data 1
Data dictionary of the assessment criteria terms used in the DIALib-QC tool.

Supplementary Data 2
DIALib-QC assessment report of eleven libraries. In all libraries, RT minimum (rt_min) and maximum (rt_max) are colored grey and light orange highlighting RT consistency in PeakView and Spectronaut formats, respectively. All criteria with erroneous values identified by DIALib-QC are highlighted in yellow when compared to the good set of libraries. a) SWATHAtlas-Pan-Human Library (PHL), b) In-house K562 sample specific libraries

Supplementary Data 3
MAYU assessment report for filtering the data at 1% protein FDR.

Supplementary Data 4
Performance of spectral libraries analyzed in PeakView. Identification of a) proteins and b) peptides using modified Pan Human Libraries (PHL), Identification of c) proteins and d) peptides using in-house K562 libraries.

Supplementary Data 5
Performance of spectral libraries analyzed in Spectronaut. Identification of a) proteins and b) peptides using modified Pan Human Libraries (PHL), Identification of c) proteins and d) peptides using in-house K562 libraries.
