# Supplementary material for: DIALib-QC an assessment tool for spectral libraries in data-independent acquisition proteomics
Source: Nat Commun. 2020 Oct 16;11:5251. doi: 10.1038/s41467-020-18901-y (PMC7567827; doi:10.1038/s41467-020-18901-y)
Supplement: Supplementary file 9 — Reporting Summary [file 41467_2020_18901_MOESM9_ESM.pdf]

nature research

Corresponding author(s): Dr. Robert L. Moritz

Last updated by author(s): Aug 28, 2020

Reporting Summary

Nature Research wishes to improve the reproducibility of the work that we publish. This form provides structure for consistency and transparency in reporting. For further information on Nature Research policies, see Authors & Referees and the Editorial Policy Checklist .

Statistics

For all statistical analyses, confirm that the following items are present in the figure legend, table legend, main text, or Methods section.

n/a	Confirmed	
☐	☒	The exact sample size (n) for each experimental group/condition, given as a discrete number and unit of measurement
☐	☒	A statement on whether measurements were taken from distinct samples or whether the same sample was measured repeatedly
☒	☐	The statistical test(s) used AND whether they are one- or two-sided<br>*Only common tests should be described solely by name; describe more complex techniques in the Methods section.*
☒	☐	A description of all covariates tested
☒	☐	A description of any assumptions or corrections, such as tests of normality and adjustment for multiple comparisons
☐	☒	A full description of the statistical parameters including central tendency (e.g. means) or other basic estimates (e.g. regression coefficient) AND variation (e.g. standard deviation) or associated estimates of uncertainty (e.g. confidence intervals)
☒	☐	For null hypothesis testing, the test statistic (e.g. F, t, r) with confidence intervals, effect sizes, degrees of freedom and P value noted<br>*Give P values as exact values whenever suitable.*
☒	☐	For Bayesian analysis, information on the choice of priors and Markov chain Monte Carlo settings
☒	☐	For hierarchical and complex designs, identification of the appropriate level for tests and full reporting of outcomes
☐	☒	Estimates of effect sizes (e.g. Cohen's d, Pearson's r), indicating how they were calculated

Our web collection on statistics for biologists contains articles on many of the points above.

Software and code

Policy information about availability of computer code

Data collection	MS data collection was performed on a SCIEX TripleTOF 6600. All spectral library evaluation was generated by the DIALib-QC tool. The latest version of DIALib-QC tool can be found at http://www.swathatlas.org
Data analysis	Two different DIA software tools were used in this study, Sciex PeakView and Biognosys Spectronaut, to perform the targeted data extraction of five analytical DIA replicates.

For manuscripts utilizing custom algorithms or software that are central to the research but not yet described in published literature, software must be made available to editors/reviewers. We strongly encourage code deposition in a community repository (e.g. GitHub). See the Nature Research guidelines for submitting code & software for further information.

Data

Policy information about availability of data

All manuscripts must include a data availability statement. This statement should provide the following information, where applicable:

- Accession codes, unique identifiers, or web links for publicly available datasets
- A list of figures that have associated raw data
- A description of any restrictions on data availability

All raw and processed data is available via the community repository ProteomeXchange.

Field-specific reporting

Please select the one below that is the best fit for your research. If you are not sure, read the appropriate sections before making your selection.

☒ Life sciences ☐ Behavioural & social sciences ☐ Ecological, evolutionary & environmental sciences

Life sciences study design

All studies must disclose on these points even when the disclosure is negative.

Sample size	For DIA experiments involving spectral library evaluation, five analytical replicates (n=5) of the K-562 cell pellet digest were measured for statistical confidence.
Data exclusions	Data were not excluded from analysis
Replication	All replication attempts were successful and included in the analysis. Replicates were performed by repeating the experiment on the same cell line.
Randomization	Not relevant, since there are no experimental groups involved in this study.
Blinding	No Blinding was not performed as not applicable for the analysis for the 5 replicates in this study.

Reporting for specific materials, systems and methods

We require information from authors about some types of materials, experimental systems and methods used in many studies. Here, indicate whether each material, system or method listed is relevant to your study. If you are not sure if a list item applies to your research, read the appropriate section before selecting a response.

Materials & experimental systems

n/a	Involved in the study	
☒	☐	Antibodies
☐	☒	Eukaryotic cell lines
☒	☐	Palaeontology
☒	☐	Animals and other organisms
☒	☐	Human research participants
☒	☐	Clinical data

Methods

n/a	Involved in the study	
☒	☐	ChIP-seq
☒	☐	Flow cytometry
☒	☐	MRI-based neuroimaging

Eukaryotic cell lines

Policy information about cell lines

Cell line source(s)	K-562 (CCL-243 human bone marrow myeloid leukemia) cell line was obtained from ATCC. The continuous cell line K-562 was established by Lozzio and Lozzio (Blood 45: 321-334, 1975. PubMed: 163658) from the pleural effusion of a 53-year-old female with chronic myelogenous leukemia in terminal blast crises.
Authentication	No additional authentication performed on source cell line. ATCC verifies the cell line to be correct through their verification process available at https://www.atcc.org/products/all/CCL-243.aspx#generalinformation
Mycoplasma contamination	The K-562 cell line tested negative for Mycoplasma contamination during culture for this project.
Commonly misidentified lines (See ICLAC register)	The K-562 cell line used for this project is not listed in the ICLAC database as contaminated. Other cell lines listed in the ICLAC database do show they can be contaminated with K-562.
