# Supplementary material for: DIALib-QC an assessment tool for spectral libraries in data-independent acquisition proteomics
Source: Nat Commun. 2020 Oct 16;11:5251. doi: 10.1038/s41467-020-18901-y (PMC7567827; doi:10.1038/s41467-020-18901-y)
Supplement: Supplementary file 2 — Peer Review File [file 41467_2020_18901_MOESM2_ESM.pdf]

Reviewer #1 (Remarks to the Author):

The manuscript by Midha et al. describes a software program (DIALib-QC) for quality assessment of spectral libraries for DIA analysis. The tool provides some useful quality checks of input spectral libraries, and a routine use of such tool could potentially lead to improved analysis of DIA data. However, the manuscript is limited in scope and technical in nature, targeting more advanced proteomics users. Specific comments:

Major comments:
1. It is not clear how broad the user base would be, as there are many pipelines and tools in use, and some of them already have at least some QC tools. Even more importantly, other than detecting the potential problems, the tool does not provide any means to improve the libraries. For example, if the tool detects that there are some entries that have q1 and q3 ions in close proximity (which may negatively affect the accuracy of quantification), how would that help the user? DIALib-QC does not correct the library to remove questionable entries, and it does not provide any guidance on how to fix the library. I think the authors have an opportunity to extend their tool to improve the spectral libraries, not just perform a QC.

2. The tool is applied to perform QC of conventional libraries built using DDA data. Many recent DIA studies are using other ways to build the libraries, e.g. directly from DIA (as in DIA-Umpire or Spectronaut), or using hybrid libraries built from DDA and DIA data. The libraries can be predicted insilico using deep learning (e.g. Prosit server), and optionally corrected using empirical gas phase fractionation (GPF) narrow window DIA data. The authors need to explore where their tool fits among this growing number of workflows used to build spectral libraries for DIA analysis.

3. One of the tests performed in the manuscript is to introduce a bias in m/z values for q1 and q3 values in the library. I do not understand why it would be particularly relevant. This would matter only if the libraries were build using experimental m/z values from DDA runs. However, to my knowledge, most DIA libraries have theoretical m/z values. And then the experimental DIA data is calibrated to correct mass errors in the DIA data. So this analysis, as presented, does not seem to be particularly relevant or interesting.

Minor comment:

4. The terminology q1 and q3 seem to be used more frequently in targeted SRM/MRM literature. In DIA studies, at least in the proteomics domain, I think it is more common to use precursor ions and fragment ions instead of q1 and q3.

Reviewer #2 (Remarks to the Author):

Midha et al describe a tool for quality assessment of libraries for use in DIA data analysis and demonstrate its utility using simulated problem libraries. This is an area that has been somewhat overlooked and the tool is a welcome addition. The tool is provided by a user friendly web interface and standalone. The report is concisely written (appreciated) and reasonably well illustrated. I would suggest the report could be published after some relatively minor revisions, especially surrounding language and clarity regarding decoys in 2 sections and perhaps some richer plots.

1. The authors state that FDR methods 'typically assume equal numbers of target and decoy assays' and cite the Ludwig tutorial DIA tutorial paper. They also state that 'Libraries containing unequal numbers of targets and decoys introduce bias in the target/decoy ratio, which can lead to false identifications.'

I don't think that this is actually the case. Software such as openswath, skyline, spectronaut (we guess about spectronaut, although who knows) that use the mprophet type approach that is based on the Storey-Tibshirani method do not rely on naive decoy counting. Rather they model the decoy score distribution (empirical null) in order to generate p-values for all targets that are later converted to q-values. This procedure is actually somewhat insensitive to the number of decoys as long as there are enough to create a robust model of the distibution. I looked in the Ludwig paper the authors cite and I don't see a mention of keeping the number of decoys and targets equal (they do mention keeping the number of fragment ions consistent between target and decoy peptides but this is differet).
This does not mean that its not useful to know the number of decoys versus targets in the library. It is! But I would suggest that the justification is rephrased. Its probably still safest to keep the target and decoy numbers roughly in the same range but the point is overstated by the authors I think.

2. I struggled to understand the discussion of decoys in the results (lines 120-127). After several re-readings I think that I now understand that the 'DECOYS' the authors refer to in this section are decoys from the DDA search (as opposed to decoys that were generated for the DIA data analysis). I assume they mean that they are then running Peakview and Spectronaut in modes where they generate their own decoys for DIA analysis. Is this correct? Because their tool only identifies 0.1-0.4% decoys (sup table 1). If so, the authors need to distinguish more clearly between these cases. Similarly, they need to make this clarification in the corresponding methods section. If I don't understand this please correct, but I found these sections very difficult to parse.
They also report the number of decoys (again, presumably leftover from the DDA analysis) that they find in the DIA analysis for peakview and spectronaut. Here they need to distiguish whether these are of the mixed variety where a peptide maps to both a target and a decoy (unproblematic) or just to a decoy.
While, peakview and spectronaut can generate decoys on the fly for DIA analysis, other tools (e.g. openswath) cannot. My assumption was that the decoy count function of the tool was check the fraction of decoys generated as part of the DIA library generation (as I think the authors suggest in lines 71-74 -- see comment 1) but this later section seems to assume the operator is testing a library prior to DIA decoy generation. This is too confusing.
One way they could help to distiguish would be to make a suggestion that the user has different decoy strings for the DDA search and for the DIA analysis in order to keep them separate. Then they could allow to specify the different decoy strings in their tool which would allow their separate evaluation (for what are 2 importantly different concepts).

3. The table output is okay but could be improved with some graphs because almost all output from their tool is numberical. Some values are expressed with summuries and could benefit from histograms or bar plots or whatever (i.e. average peptide length or peptides per protein or average number of q3 per q1, etc, etc). This may not be essential at this time but I think would be a welcome addition especially for the web interface.

Response to the Reviewers

We greatly appreciate the time taken by the Reviewers for the comments and suggestions to improve out manuscript. Please find following our point-by-point responses below. The text in blue are comments and questions provided by the Reviewers and our responses are immediately following each comment in black text.

Reply to Reviewer 1's queries

We thank the reviewer for their time, the positive feedback, and useful comments to our manuscript. Addressing these comments has improved our manuscript in meaningful ways. We made several changes in the DIALib-QC tool to provide the opportunity to assess and now even correct a spectral ion library via our tools user-friendly interface. In addition, we now also provide various graphical plots of the results that can be downloaded along with the tabular assessment report. Below we respond to each comment point-by-point.

Reviewer #1 (Remarks to the Author):

The manuscript by Midha et al. describes a software program (DIALib-QC) for quality assessment of spectral libraries for DIA analysis. The tool provides some useful quality checks of input spectral libraries, and a routine use of such tool could potentially lead to improved analysis of DIA data. However, the manuscript is limited in scope and technical in nature, targeting more advanced proteomics users.

We thank the reviewer for these comments. In the revised article, we have substantially improved the tool by 1) making the interactive DIALib-QC assessment tool more user-friendly for non-

advanced users (detailed readme file with installation instructions, usage flow chart, and demo files of input library formats, expected output and associated commands are provided); 2) providing a tabular QC report along with a single page graphical summary plot of various library characteristics for ease of understanding (downloadable); 3) extending the DIALib-QC capabilities to repair and filter ion libraries; 4) added the functionality to assess *in-silico* predicted spectral libraries generated using Prosit server and its usage instructions are also provided at the 'DIALib-QC 1.1 (download)' page at http://www.swathatlas.org/DIALibQC.php.

We expect that the much improved tool including the newly added single-page graphical display of the results along with a concisely written manuscript, well-illustrated schemes (as pointed out by the reviewers), and detailed instructions on the webpage will certainly cater attention of non-expert users and facilitate the use of DIALib-QC tool to assess and improve their libraries.

Specific comments:

It is not clear how broad the user base would be, as there are many pipelines and tools in use, and some of them already have at least some QC tools.

To address the concerns of the reviewer, although that there are several tools and pipelines to generate and merge spectral assay libraries, and some can perform limited rudimentary QC steps, they are confined to a particular analysis pipeline, format or vendor, limited in scope, and none of them allow to correct a library, our much improved tool addresses each of these points and provides a new system for quality control and repair which undoubtedly will garner a large user base. DIALib-QC performs an in-depth assessment and provides valuable insights of five major categories (total 62 criteria), out of which the correctness criterion is useful in generating error-

free libraries by removing problematic assays. All these assessment features are extremely relevant to all users regardless of pipeline, whether generating in-house libraries or using public repository libraries. Ultimately, this tool will cater the attention of all DIA researchers, using Spectronaut, OpenSWATH, PeakView and virtually all in-house DIA analysis tools.

We have added relevant sentences in the introduction section of the manuscript for better clarity:

On page 3 (Introduction): "Several tools and pipelines generate and merge spectral assay libraries, but few perform library quality assessment and are limited to a particular analysis pipeline, format or vendor, or are limited in scope, and none are able to correct a library. One such reported tool, SWATHXtend[9], applies certain quality filters to generate clean hybrid libraries but does not evaluate spectral ion libraries for correctness. DIALib-QC performs an in-depth assessment, providing valuable characteristics of a library and also enables users to correct and generate an error-free library by eliminating problematic assays. These DIALib-QC features are extremely relevant to all DIA users regardless of the pipeline used (whether generating in-house libraries or using public repository libraries), library formats and DIA analysis tools."

Even more importantly, other than detecting the potential problems, the tool does not provide any means to improve the libraries. For example, if the tool detects that there are some entries that have q1 and q3 ions in close proximity (which may negatively affect the accuracy of quantification), how would that help the user? DIALib-QC does not correct the library to remove questionable entries, and it does not provide any guidance on how to fix the library. I think the authors have an opportunity to extend their tool to improve the spectral libraries, not just perform a QC.

We highly appreciate the reviewer's suggestion and thank you for raising this point in our manuscript. We have now extended the tool and added such capabilities. In the new version of DIALib-QC, the tool provides means of repairing the spectral ion library for mass correction by recomputing theoretical masses if incorrect masses are detected. Moreover, conflict assays can be filtered by separating the problematic assays from the good assays. This will help the user to generate clean spectral libraries with no mass error and finally by removing questionable conflict entries from the library prior to DIA analysis ensuring correct peptide quantitation is achieved. To guide the user, we have updated the usage instructions of DIALib-QC with demo files on the download page 'DIALib-QC 1.1 (download)' at http://www.swathatlas.org/DIALibQC.php.

To address these updates, we have made several relevant changes in the revised manuscript text, figures and its legends as well as supplementary information as follows:

On page 3 (Introduction): "Further, we show DIALib-QC and its functionality to repair an ion library by correcting mass errors (recomputing to theoretical *m/z* values) and removing conflict assays to generate a clean and correct spectral library for DIA analysis."

On page 3 (Results): "DIALib-QC inspects 62 criteria of a library and provides valuable insights of its composition in a tabulated form."

On page 4 (Results): "2) Mass Accuracy: DIALib-QC compares precursor and fragment ion *m/z's* to theoretical values and provides the average delta mass, highlighting the accuracy of masses used in the library. The total number of these erroneous assays along with assays that cannot be verified with the input SWATH definition file (see category 4 below and **Methods**) are reported as the criterion 'problem assays'. Such assays can be further explored in the DIALib-QC ion library repair feature to generate a clean library, free from mass errors and conflict assays."

On page 8 (Results): "To demonstrate the use of DIALib-QC in constructing an error-free library, it's 'repairing' option was applied on the 'q3bad' library. This option recomputes the theoretical *m/z* values of the fragment ions, generating a mass corrected library ('q3bad_mz_corrected') (**Supplementary Data 2**). In this corrected library, DIALib-QC reported no average mass difference in the fragment ions but identified five conflict assays (**Supplementary Data 2**). These assays became conflict assays after the mass correction step as they now fall in the SWATH precursor window and were filtered sequentially by running the DIALib-QC 'filter conflict assay' option. Two resultant final libraries were exported, one consisting of all conflict assays for inspection, and the other consisting of error-free assays ('q3bad_mz_corrected_clean' library) for use in DIA analysis. Additionally, characteristics of the error-free library ('q3bad_mz_corrected_clean') are graphically represented in **Supplementary Fig. 9**. DIALib-QC assessment of the error-free library showed no problem assays (**Supplementary Data 2**) and hence mimics its 'good' counterpart, as an ideal spectral library for the downstream DIA/SWATH analysis."

On page 14 (Methods): We added two new libraries to describe the DIALib-QC repair and filter options as: "*Library 6: K562 'q3bad_mz_corrected' (consensus spectra + corrected theoretical fragment ion m/z values)*

This library was generated from Library 4 ('q3bad') by correcting the observed with to theoretical fragment ion *m/z* values using the DIALib-QC 'repairing' option. The final library generated has no mass error.

Library 7: K562 'q3bad_mz_corrected_clean' (consensus spectra + corrected theoretical fragment ion m/z values + removed conflict assays)

This library was generated from Library 6 by removing the conflict assays identified in reference to the SWATH definition file by using the ‘filter conflict assays’ option of DIALib-QC. The resultant library is free from any mass errors and conflict assays.”

On pages 19-20: We revised Figure 1 and included the following in its legend, “The workflow in black solid lines shows all functions to analyze an assay library and generates an assessment report and graphical summary plots. Mass Correction (green solid lines) and Filtering Conflict Assays (orange solid lines) functions are tandem features that generate a new library. Using the Mass Correction parameter results into a ‘Mass Corrected Library’ while using the Filter Conflict Assays parameter segregates the query library into a ‘Problem’ (containing only conflict assays) and a ‘Clean’ (error-free) library. The Proteome Mapping file (purple dashed line) and SWATH Definition file (blue dashed line) are optional input files which provide information related to peptide/protein characteristics and help to asses conflict assays, respectively.”

On page 3 (Supplementary Information): We have added the “q3_mz_corrected” and “q3_mz_corrected_clean” libraries information in the Supplementary Figure 1 and updated its legend.

Supplementary Data 2 (Excel file): Revised DIALib-QC reports of nine existing libraries and two newly added reports for ‘q3bad_mz_corrected’ and ‘q3bad_mz_corrected_clean’ libraries are provided in the Supplementary Data 2.

Additional minor text changes were made to the Abstract (page 2), Introduction (pages 2 and 5) and Conclusion (page 8).

2. The tool is applied to perform QC of conventional libraries built using DDA data. Many recent DIA studies are using other ways to build the libraries, e.g. directly from DIA (as in DIA-Umpire or Spectronaut), or using hybrid libraries built from DDA and DIA data. The libraries can be predicted insilico using deep learning (e.g. Prosit server), and optionally corrected using empirical gas phase fractionation (GPF) narrow window DIA data. The authors need to explore where their tool fits among this growing number of workflows used to build spectral libraries for DIA analysis.

We thank the reviewer for their insight and agree that DIA library generation can be performed from many different workflows. It is precisely this reason that DIALib-QC is so important, and why we recommend library assessment to be performed prior to its use. To directly address this issue, we have extended the scope of DIALib-QC capabilities and illustrate how one would assess and repair in-silico predicted spectral libraries generated using the Prosit server. Instructions for users on how to use the DIALib-QC tool to assess libraries generated by Prosit server are made available on the DIALib-QC download page at www.swathatlas.org (http://www.swathatlas.org/DIALibQC.php) along with a demo input library file and expected output.

We have made related changes in the revised manuscript text and figures as follows:

On page 2 (Introduction): "DIALib-QC reports a tabular and graphical summary of common DIA library formats including OpenSWATH, Spectronaut, PeakView and Prosit (generic text, Spectronaut compatible)."

On page 15 (Methods): "DIALib-QC provides assessment reports for all DDA derived libraries in both library formats, PeakView and Spectronaut (**Supplementary Data 2**). In addition, DIALib-QC is also compatible with DIA derived libraries as described in Supplementary Note 1."

On page 18 (Supplementary Information): Supplementary Note 1: "DIALib-QC is fully capable to evaluate DIA derived libraries constructed using untargeted workflows in Spectronaut and DIA-Umpire[1]. The tool supports Spectronaut format libraries regardless of, if it is built from DDA or DIA data. Similarly, DIA-Umpire analysis can be used to generate a library directly from DIA data using the SpectraST pipeline, which produces library formats (OpenSWATH and PeakView) compatible with DIALib-QC. In case of other formats, users can transform their library to formats compatible with DIALib-QC for its assessment and repair.

1. Tsou, C. C. *et al.* DIA-Umpire: Comprehensive computational framework for data-independent acquisition proteomics. *Nat. Methods* **12**, 258–264 (2015)."

On page 19 (Revised Figure 1): Updated to include Prosit.

3. One of the tests performed in the manuscript is to introduce a bias in m/z values for q1 and q3 values in the library. I do not understand why it would be particularly relevant. This would matter only if the libraries were build using experimental m/z values from DDA runs. However, to my knowledge, most DIA libraries have theoretical m/z values. And then the experimental DIA data is calibrated to correct mass errors in the DIA data. So this analysis, as presented, does not seem to be particularly relevant or interesting.

We share the concerns of the reviewer that the relevance of *m/z* values is diminished if all users were aware of the need to ensure theoretical *m/z* values, and as most but not all of the library generation tools provide theoretical *m/z* values. However, the danger of such assumptions that all

users do not make mistakes is too hard a stance to take since improperly calculated *m/z* values in a library will have deleterious effects on DIA analysis and subsequent downstream conclusions being reported, even with internal calibration of the DIA software (page 6 and Figure 2a). We chose this example because it is most often taken for granted, and is therefore particularly relevant and interesting. To our knowledge, commonly-used open source pipelines (such as SpectaST) by default report empirical *m/z* values, which can trip up both experienced and novice users of DIA methods. Furthermore, given that DIA is still a nascent and rapidly developing niche in MS, analysis tools are constantly being developed and evolved and it is not safe to assume they are all equal in their creation and use of DIA libraries. We provide a poignant example on page 6 and in Figure 2, illustrating that calibration to correct mass errors in the DIA data is not a common feature shared by all pipelines, and, in fact, did not exist at all in some of the most widely used tools in the field. Furthermore, assuming mass calibration, if it exists, would solve the problem still resulted in a dramatic (30%) decrease in peptides identified and quantified (page 6 and Supplementary Figure 4). Therefore, we feel this example is an excellent illustration of the importance of using DIALib-QC to assess spectral libraries (and subsequently correct them if needed).

Minor comment:

4. The terminology q1 and q3 seem to be used more frequently in targeted SRM/MRM literature. In DIA studies, at least in the proteomics domain, I think it is more common to use precursor ions and fragment ions instead of q1 and q3.

We thank the reviewer for their views on mass terminology highlighted this in the manuscript. For clarity, we duly made these changes to the text to provide an agreed nomenclature of precursor

ions and fragment ions in place of q1 and q3, respectively in the revised article and also in the QC report generated by the DIALib-QC tool.

Answers to Reviewer 2's queries

Reviewer #2 (Remarks to the Author):

Midha et al describe a tool for quality assessment of libraries for use in DIA data analysis and demonstrate its utility using simulated problem libraries. This is an area that has been somewhat overlooked and the tool is a welcome addition. The tool is provided by a user friendly web interface and standalone. The report is concisely written (appreciated) and reasonably well illustrated. I would suggest the report could be published after some relatively minor revisions, especially surrounding language and clarity regarding decoys in 2 sections and perhaps some richer plots.

We sincerely thank the reviewer for their time and effort in providing valuable recommendations to improve the manuscript. We have addressed the major concerns by defining 'decoy assays' and 'decoy peptides' in context of DDA searching and DIA analysis, and also provide graphical plots (downloadable) along with tabular QC report of library characteristics.

1. The authors state that FDR methods 'typically assume equal numbers of target and decoy assays' and cite the Ludwig tutorial DIA tutorial paper. They also state that 'Libraries containing unequal numbers of targets and decoys introduce bias in the target/decoy ratio, which can lead to false identifications.'

I don't think that this is actually the case. Software such as openswath, skyline, spectronaut (we guess about spectronaut, although who knows) that use the mprophet type approach that is based on the Storey-Tibshirani method do not rely on naive decoy counting. Rather they model the decoy score distribution (empirical null) in order to generate p-values for all targets that are later converted to q-values. This procedure is actually somewhat insensitive to the number of decoys as

long as there are enough to create a robust model of the distribution. I looked in the Ludwig paper the authors cite and I don't see a mention of keeping the number of decoys and targets equal (they do mention keeping the number of fragment ions consistent between target and decoy peptides but this is differet).

This does not mean that its not useful to know the number of decoys versus targets in the library. It is! But I would suggest that the justification is rephrased. Its probably still safest to keep the target and decoy numbers roughly in the same range but the point is overstated by the authors I think.

We thank the reviewer for pointing this out. As suggested, we have rephrased the paragraph and replaced the Ludwig *et al.* reference with Reiter, L. *et al.* MProphet: Automated data processing and statistical validation for large-scale SRM experiments. *Nat. Methods* **8**, 430–435 (2011) (refernce # 12 in the revised manuscript). The text now reads:

On page 4 (Results): "1) Target/Decoy percentage: DIALib-QC reports the percentage of target, mixed (map to both target and decoy) and decoy assays present in the library. Decoy assays are assays to peptides that are not present in the target proteome. These assays could be in-silico derived, spiked into the library or inherited from DDA analysis during library generation. Some DIA pipelines require decoy assays for FDR estimation using a target-decoy approach, provided sufficient decoy assays are present for robust error modeling[12]."

2. I struggled to understand the discussion of decoys in the results (lines 120-127). After several re-readings I think that I now understand that the 'DECOYS' the authors refer to in this section are decoys from the DDA search (as opposed to decoys that were generated for the DIA data analysis).

I assume they mean that they are then running Peakview and Spectronaut in modes where they generate their own decoys for DIA analysis. Is this correct? Because their tool only identifies 0.1-0.4% decoys (sup table 1). If so, the authors need to distinguish more clearly between these cases. Similarly, they need to make this clarification in the corresponding methods section. If I don't understand this please correct, but I found these sections very difficult to parse.

We thank the reviewer for suggesting improvements in clarity and agree that the word decoy is commonly used in different contexts in many aspects of MS data analysis. Yes, it is correct that the decoy assays present in the 'base' library are inherited from the DDA analysis during the library generation process. It is also correct that both PeakView and Spectronaut generate their own decoys on the fly to estimate the FDR. The 'decoy assay' information of 0.1-0.4% provided by the DIALib-QC tool (Supplementary Data 2) exclusively belongs to the "base" spectral library (these are carried forward from the DDA experiment) and does not correspond to the 'decoy peptides' obtained in the DIA analysis. We have endeavored to clearly define the contexts in which we use the word. To summarize, we now clearly define 'decoy assays' as any assay belonging to a non-existent peptide in the DIA library, that were left over from the DDA analysis, and 'decoy peptides' as the peptide sequences identified from decoy assays during DIA analysis.

Details of where we made these changes are:

On page 4 (Results): As addressed in the previous response.

On page 10 (Methods): "The datasets were searched with Comet[15] (2017.01r1) against the full non-redundant, canonical human proteome from UniProtKB/Swiss-Prot (2018_07) with 20,270 ORFs and appended contaminants, shuffled decoy sequences and iRT peptides (Biognosys)."

On pages 12-13 (Methods): “Decoy assays are assays to peptides that do not exist in the human (target) proteome and are inherited from the DDA analysis during the library generation step. Peptide sequences identified from these decoy assays (referred to as decoy peptides in DIA analysis) are therefore known to be false. Further, the library was limited to the top6 fragments per precursor without enabling the SWATH verification which kept the conflict assays in the library.”

They also report the number of decoys (again, presumably leftover from the DDA analysis) that they find in the DIA analysis for peakview and spectronaut. Here they need to distiguish whether these are of the mixed variety where a peptide maps to both a target and a decoy (unproblematic) or just to a decoy.

In the revised manuscript, we do not provide the decoy peptide information from the DIA analysis and do not show the repercussions of library decoy assays on DIA results. As these decoy assays were small in proportion in the ‘base’ library, which were not sufficient to perform the entrapment strategy in the DIA analysis. Although, we emphasize that the information of decoy assays in the library reported by DIALib-QC is crucial in the management of decoys for the adequate DIA analysis. Further, the effect of entrapment analysis on estimating the realized FDR by the DIA tools is beyond the scope of this manuscript. In lieu of this, we have added the following text as below:

On pages 6-7 (Results): “DIALib-QC identified the small fraction (0.1%) of decoy assays in the ‘base’ library, that were left over from the DDA analysis during the library generation process. These assays were therefore removed in the ‘conflict’ and ‘good’ libraries and thus decoy numbers were confirmed as zero by DIALib-QC (**Supplementary Data 2**). However, *in-silico* decoy assays

are often appended to the library in tools such as OpenSWATH for false discovery rate (FDR) estimation. For DIA tools (such as Spectronaut and PeakView) that apply their own mechanism to estimate the FDR, *in silico* decoy assays can be added to the library to perform an entrapment analysis[13]. During entrapment analysis, the false positive identifications of decoy peptides are estimated by matching to these decoy assays following DIA analysis, which can be used to calculate a realized FDR that is compared to the FDR estimate provided by the software. In either case, knowledge of the number of decoy assays is essential to determine if sufficient and not excessive assays are available to perform adequate DIA analysis."

On page 18 (Reference): We have replaced the previous reference number 13 with the following: "Feng, X. dong *et al.* Using the entrapment sequence method as a standard to evaluate key steps of proteomics data analysis process. *BMC Genomics* **18**, 1–9 (2017)."

On pages 21-22 (Figures): Figure 2 and its legend have been updated.

While, peakview and spectronaut can generate decoys on the fly for DIA analysis, other tools (e.g. openswath) cannot. My assumption was that the decoy count function of the tool was check the fraction of decoys generated as part of the DIA library generation (as I think the authors suggest in lines 71-74 -- see comment 1) but this later section seems to assume the operator is testing a library prior to DIA decoy generation. This is too confusing.

We thank the reviewer for pointing this out. We have rephrased sentences to avoid confusion as addressed in the previous responses (revised manuscript page numbers 4 and 7).

One way they could help to distinguish would be to make a suggestion that the user has different decoy strings for the DDA search and for the DIA analysis in order to keep them separate. Then they could allow to specify the different decoy strings in their tool which would allow their separate evaluation (for what are 2 importantly different concepts).

We thank the reviewer for their valuable suggestion. The text has been clarified as described above, although the text's new phrasing does not require such distinction, allowing the user to decide their preferred decoy nomenclature.

3. The table output is okay but could be improved with some graphs because almost all output from their tool is numberical. Some values are expressed with summuries and could benefit from histograms or bar plots or whatever (i.e. average peptide length or peptides per protein or average number of q3 per q1, etc, etc). This may not be essential at this time but I think would be a welcome addition especially for the web interface.

With many thanks to the reviewer for this suggestion, we believe our changes, not only in the manuscript but also the tool, will address the poor perception of the result presentation and the reviewer will be satisfied with the outcome. We have now implemented the generation of nine plots summarizing several of the main library characteristics that are assessed with DIALib-QC. These DIALib-QC plots are compiled into a single pdf page, downloadable by the user along with the tabular results at both the web interface and standalone version, and could be used by authors to submit alongside their supplementary material on papers describing new DIA libraries.

The relevant text is added in the following sections:

On page 2 (Introduction): “DIALib-QC reports a tabular and graphical summary of common DIA library formats including OpenSWATH, Spectronaut, PeakView and Prosit (generic text, Spectronaut compatible)”

On page 8 (Results): “Additionally, characteristics of the error-free library (‘q3bad_mz_corrected_clean’) are graphically represented in **Supplementary Fig. 9**.”

On pages 19-20 (Figures): We have revised Figure 1 and its legend.

On page 3 (Supplementary Information): Revised Supplementary Figure 1 by adding DIALib-QC graph plot file.

On pages 16-17 (Supplementary Information): Added a new Supplementary Figure 9 and its legend.

REVIEWERS' COMMENTS:

Reviewer #1 (Remarks to the Author):

The authors addressed my comments, and importantly, added new functionality to repair the libraries. I have no additional comments.

Reviewer #2 (Remarks to the Author):

The authors have addressed the comments thoroughly. I would recommend to accept at this stage.
